# Diffuse alveolar damage patterns reflect the immunological and molecular heterogeneity in fatal COVID-19

**DOI:** 10.1016/j.ebiom.2022.104229

**Published:** 2022-08-24

**Authors:** Jonas S. Erjefält, Natália de Souza Xavier Costa, Jimmie Jönsson, Olga Cozzolino, Katia Cristina Dantas, Carl-Magnus Clausson, Premkumar Siddhuraj, Caroline Lindö, Manar Alyamani, Suzete Cleusa Ferreira Spina Lombardi, Alfredo Mendroni Júnior, Leila Antonangelo, Caroline Silvério Faria, Amaro Nunes Duarte-Neto, Renata Aparecida de Almeida Monteiro, João Renato Rebello Pinho, Michele Soares Gomes-Gouvêa, Roberta Verciano Pereira, Jhonatas Sirino Monteiro, João Carlos Setubal, Ellen Pierre de Oliveira, Jair Theodoro Filho, Caroline Sanden, Jamie M. Orengo, Matthew A. Sleeman, Luiz Fernando Ferraz da Silva, Paulo Hilário Nascimento Saldiva, Marisa Dolhnikoff, Thais Mauad

**Affiliations:** a Unit of Airway inflammation, Department of Experimental Medicine Sciences, Lund University, Sweden; b Department of Allergology and Respiratory Medicine, Lund University, Sweden; c Departamento de Patologia, LIM-05 Laboratório de Patologia Ambiental, Faculdade de Medicina da Universidade de São Paulo, São Paulo, Brazil; d Medetect AB, Lund, Sweden; e Divisão de Pesquisa & Medicina Transfusional, Fundação Pró-Sangue Hemocentro de São Paulo, São Paulo, Brazil; f Laboratório Investigação Médica em Patogênese e Terapia dirigida em Onco-Imuno-Hematologia (LIM-31), Departamento de Hematologia, Hospital das Clínicas HCFMUSP, Faculdade de Medicina, Universidade de São Paulo, São Paulo, Brazil; g Laboratório de Investigação Médica (LIM03), Hospital das Clínicas HCFMUSP, Faculdade de Medicina, Universidade de São Paulo, São Paulo, Brazil; h Divisão de Patologia Clínica – Departamento de Patologia, Hospital das Clínicas HCFMUSP, Faculdade de Medicina, Universidade de São Paulo, São Paulo, Brazil; i Hospital Israelita Albert Einstein, São Paulo, Brazil; j Departamento de Gastroenterologia (LIM-07), Faculdade de Medicina da Universidade de São Paulo, São Paulo, Brazil; k Departamento de Bioquímica, Instituto de Química Universidade de São Paulo, São Paulo, Brazil; l Departamento de Cardiopneumologia, Instituto do Coração, Faculdade de Medicina da Universidade de São Paulo, São Paulo, Brazil; m Regeneron Pharmaceuticals, Tarrytown, NY, USA; n Serviço de Verificação de Óbitos da Capital, Universidade de São Paulo, São Paulo, Brazil

**Keywords:** COVID-19, SARS-Cov-2, Immunopathology, Diffuse alveolar damage, Autopsy

## Abstract

**Background:**

Severe COVID-19 lung disease exhibits a high degree of spatial and temporal heterogeneity, with different histological features coexisting within a single individual. It is important to capture the disease complexity to support patient management and treatment strategies. We provide spatially decoded analyses on the immunopathology of diffuse alveolar damage (DAD) patterns and factors that modulate immune and structural changes in fatal COVID-19.

**Methods:**

We spatially quantified the immune and structural cells in exudative, intermediate, and advanced DAD through multiplex immunohistochemistry in autopsy lung tissue of 18 COVID-19 patients. Cytokine profiling, viral, bacteria, and fungi detection, and transcriptome analyses were performed.

**Findings:**

Spatial DAD progression was associated with expansion of immune cells, macrophages, CD8+ T cells, fibroblasts, and (lymph)angiogenesis. Viral load correlated positively with exudative DAD and negatively with disease/hospital length. In all cases, enteric bacteria were isolated, and *Candida parapsilosis* in eight cases. Cytokines correlated mainly with macrophages and CD8+T cells. Pro-coagulation and acute repair were enriched pathways in exudative DAD whereas intermediate/advanced DAD had a molecular profile of elevated humoral and innate immune responses and extracellular matrix production.

**Interpretation:**

Unraveling the spatial and molecular immunopathology of COVID-19 cases exposes the responses to SARS-CoV-2-induced exudative DAD and subsequent immune-modulatory and remodeling changes in proliferative/advanced DAD that occur side-by-side together with secondary infections in the lungs. These complex features have important implications for disease management and the development of novel treatments.

**Funding:**

CNPq, Bill and Melinda Gates Foundation, HC-Convida, FAPESP, Regeneron Pharmaceuticals, and the Swedish Heart & Lung Foundation.

**Research in context**

**Evidence before this study**

In severe cases of COVID-19, the lungs are subject to heterogeneous, viral-induced diffuse alveolar damage (DAD). This immunopathological picture is complicated by secondary events like non-viral infections and life-supporting management. It is important to better understand this complexity to improve disease management and optimize novel treatment strategies. Searching PubMed, we did not find studies that dissected the immunopathological and molecular features of different DAD patterns that might coexist in the same individual. Here, we present a spatially resolved description of the immune and structural cell patterns within DAD patterns using multiplex immunohistochemistry, artificial intelligence, and spatial statistics, coupled with analyses of viral load, secondary pathogens, lung cytokines, and transcriptomic analysis.

**Added value of this study**

Using a broad combination of different techniques, we detailed for the first time the immunopathological composition of the heterogeneous COVID-19 lung lesions that coexist in the same individual. In the exudative DAD pattern, epithelial and endothelial denudation with less infiltrating immune cells and higher viral load prevailed, with upregulated genes related to acute responses and thrombogenesis. In the intermediate/advanced DAD, the proliferation of epithelial cells together with increased dendritic cells seemed to drive a macrophagic and lymphocytic response, together with matrix remodeling and (lymph)angiogenesis, along with several upregulated genes related to humoral immune responses and extracellular matrix organization.

**Implications of all the available evidence**

Our demonstration of coexisting spatially distinct immunopathological and molecular cycles of DAD progression within the lungs of fatal COVID-19 exposes the magnitude of the complexity that must be considered when interpreting the hitherto published data in the field and designing improved treatment strategies for critically ill COVID-19 patients.

Alt-text: Unlabelled box

## Introduction

The lungs are the target organs of SARS-CoV-2 infection causing severe/critical disease in 20% of symptomatic individuals.^1^ The COVID-19 pandemic has so far resulted in millions of cases of severe acute respiratory disease worldwide, a proportion not seen since the Spanish flu.^2^ A significant body of literature on lung pathology has been generated so far, in contrast to other respiratory viral diseases.

Several reports have described the pathology of lung involvement in SARS-CoV-2 infection, from descriptive pathology to single-cell analysis studies, frequently using autopsy material.^3^ In its severe form, COVID-19 induces diffuse alveolar damage (DAD) with a high frequency of thrombosis, associated with other injuries, such as secondary bronchopneumonia and infarction.^3^

Immunological data and pharmacological trials so far suggest that COVID-19 lung disease is not driven by one specific cell population alone or one class of mediators.^4^ Surprisingly, little is known about the immunopathological alterations as severe COVID-19 progresses from acute to later DAD stages. Furthermore, individual factors, secondary infections, treatment, and ventilation modes are also likely to affect the pathology and its progression over time,^5^ leading to a heterogeneous picture. Decoding immune signatures at the microenvironmental level would provide new information regarding the course of responses.

The academic hospital of Sao Paulo University is the tertiary care center for severe cases of COVID-19 in Sao Paulo. Our group has been performing minimally invasive autopsies (MIA) in COVID-19 patients, contributing to the description of COVID-19 pathology.^5^^,^^6^

To date, few studies analyzed the nature of the spatial cell arrangement during the complex and heterogeneous inflammatory/immune/molecular and structural cell alterations that take place in the lungs with severe COVID-19, coupling to the presence of the virus and secondary infectious agents in tissue. To this end, we have applied a platform for multiplex imaging complemented with the assessment of SARS-CoV-2, bacterial/fungi identification, and cytokine/transcriptomic profiling of 18 deceased patients.

## Methods

### Ethics

The National Research Ethics Commission (CONEP) approved this study (CAAE #30364720.0.0000.0068). Autopsies were performed after written informed consent by the next-of-kin from all participants.

### Study population

We included 18 adult patients who died between March and April 2020 due to COVID-19 in Brazil. During this period, there were 842 admissions due to COVID-19 in the hospitals linked to the Medical School, with 301 deaths. No vaccines were available at the time. As controls, we selected postmortem biopsies of three cases with non-viral exudative DAD and six cases of acute cardiovascular deaths with normal lung histology.

### Minimally invasive autopsy

During the study period, all autopsy procedures were suspended in Brazil, and we had the approval to perform MIA for research purposes. MIA/US protocol in COVID-19 was described previously by Duarte-Neto et al.^6^ Details on postmortem interval and lung sampling are presented in the supplement.

### Histopathology

The following histological patterns in all sampled tissue of each case were quantified and expressed as percentages: normal lung, exudative DAD, intermediate DAD, advanced DAD, bronchopneumonia, and vascular changes. The histological criteria used were the following: (a) normal lung: lung parenchyma with normal histology or minimal non-specific changes as mild oedema and congestion; (b) acute/exudative DAD: interstitial and/or intra-alveolar oedema, interstitial inflammation, variable amounts of alveolar hemorrhage and fibrin deposition, intra-alveolar hyaline membranes and type II pneumocyte hyperplasia; (c) intermediate DAD: any degree of fibroblastic proliferation within the interstitium and/or alveolar spaces, including loose aggregates of fibroblasts admixed with scattered inflammatory cells, collagen deposition, squamous metaplasia, intermingled with areas of hyaline membranes; (d) advanced DAD: the same as above, with the predominance of dense fibrotic areas and no hyaline membranes.^5^

### Multiplex immunohistochemistry (MIHC)

A platform for multiplex staining (*Additive Multiplex Labeling Cytochemistry, AMLC Platform, Medetect AB, Sweden*) using the principle of cyclic immunohistochemistry was used for simultaneous visualization of multiple leukocytes and structural cell populations within single lung sections from COVID-19 patients and control individuals. An example of image and exploration is briefly shown in Vídeo S1 (Supplementary Appendix). Method details and cell markers are outlined in the supplementary appendix and Table S1. In addition, for the identification of M1 (lysozyme)/M2 (CD206) macrophages we have used immunofluorescence double staining. The methodology is presented in the supplementary appendix.

### Artificial intelligence-based spatial heterogeneity analysis of COVID-19 cases

The spatial relationship between the multiple cell markers was analyzed by an artificial intelligence (AI)-based point pattern (i.e. x,y coordinate-based) approach^7^ as well as a marker density-based heat map/grid analysis. Briefly, to generate x,y coordinates we used the functions Distance Transform Watershed (FIJI - ImageJ) and Analyze Particles (FIJI - ImageJ) that were used as input for the CytMAP Toolbox.^7^ Then, the functions “Raster Scan Neighborhoods'' and “Classify Neighborhoods into Regions'' were applied to define different tissue regions with similar immune cell composition. The classification was done using the artificial neural networks Self Organizing Map and the number of cell constellation classes were defined as the minimum of the Davies-Bouldin function. For the density-based approaches, the digitized images were overlaid with a square raster of 1000 × 1000 pixel squares. The density of each marker was extracted from each square and the spatial correlations between cell markers was plotted as correlation matrix of different cell phenotypes using a custom MATLAB code exploiting the functions *corrcoef* and *clustergram.* See supplementary appendix for further details.

### Immune cell and structural profiling in DAD patterns of COVID-19 cases

A spatial microenvironment analysis was applied to generate immune cell signatures and structural alterations at distinct patterns of DAD. In brief, regions of interest (ROIs), each with a homogeneous DAD pattern were selected from standard H&E sections. The corresponding ROIs from the consecutive neighboring multiplex-stained sections were outlined and decoded for cell marker densities and composition using a histomic SQL raw database (Cell Community Viewer, Medetect, Lund, Sweden). Details are in the online supplementary appendix.

The generated DAD ROI data were subjected to multivariate analysis using a MATLAB-based principal component analysis (PCA) and K-means exploration to identify cluster formation in the PCA plot.^8^ The markers having the most influence in the separation of DAD-associated clusters were identified by loading information.

### *In situ* hybridization of COVID-19 cases

Visualization of SARS-CoV-2 and pan bacteria (16SrRNA) mRNA was performed through double *in situ* hybridization (ISH) using the RNAscope® Multiplex fluorescence kit V2 assay kit (#323100; Advanced Cell Diagnostics, Hayward, CA, USA) using the RNAscope® 2.5 HD Detection Reagents-RED (#322360, Advanced Cell Diagnostics) for chromogenic ISH (CISH), according to the manufacturer's instructions. Details are presented in the supplementary appendix.

### xMAP luminex cytokine multiplex assay of COVID-19 cases

Frozen tissue of one postmortem biopsy site per case of the COVID-19 patients was prepared using Bio-plex Cell Lysis Kit (Bio-rad Laboratories, Hercules, CA, USA), following the manufacturer's instructions. There was no availability of frozen tissue from controls. Cytokines and chemokines were assessed using the following kits: Bio-plex TGF-β, Bio-plex Human Chemokine (Bio-rad, Hercules, California), and Milliplex MAP Human Cytokine/Chemokine (Millipore Corp., Billerica, MA) according to the kit-specific protocols. Data were expressed in pg/g and were correlated with clinical and pathological parameters. Details are in the supplementary appendix.

### Nucleic acids extraction of COVID-19 cases

DNA and RNA were extracted from 80-µm thick cryosections. Briefly, the samples were incubated for 3h at 56°C in 500μL of lysis buffer and 100 mg/mL of proteinase K (Qiagen, Hilden, Germany). Then, DNA and RNA were extracted using the Magna Pure Compact Nucleic Acid Isolation kit (Roche Diagnostics GmbH, Mannheim, Germany), according to the manufacturer's instructions.

### Real-time reverse-transcription PCR of COVID-19 cases

The viral load of SARS-CoV-2 was assessed by an in-house real-time PCR assay that amplified part of the envelope protein gene, using conditions described in Corman et al.^9^ Details in the supplementary appendix and Table S2.

### Fungi and bacteria detection in COVID-19 cases

For the detection of pathogenic fungi, conventional nested PCR was performed with specific primers for the 28S to 18S interval regions of the rRNA gene (Table S2), using conditions as described in Fujita et al.^10^ For the detection of bacteria pathogenic to humans, conventional PCR was performed with specific primers for the 16S rDNA (Table S2). Samples positive for fungi and bacteria were submitted to sequencing analysis according to the Sanger method. Details in the supplementary appendix.

### Transcriptomics

Twelve out of the 18 COVID-19 cases had adequate RNA quality of the frozen lung tissue to perform the transcriptomic analyses. Four control cases of individuals who died due to non-pulmonary causes and had frozen tissue of adequate quality were selected. Characteristics of the control individuals used for this transcriptomic analysis are presented in Table S3. The methodology is fully described in the supplementary appendix. The transcriptomic data discussed in this publication have been deposited in NCBI's Gene Expression Omnibus (accession number GSE205099).

### Statistical analysis

SPSS 21 software (SPSS Inc/IBM Chicago, USA) was used for the statistical analyses. For each variable and group, we calculated mean, median, standard error, standard deviation, and interquartile range. Data distribution was assessed by the Shapiro-Wilk normality test. We performed the non-parametric Kruskal–Wallis test, followed by the Bonferroni post hoc test to compare the patients’ and DAD groups. In addition, we performed the Spearman correlation test between variables; coefficients (r) were considered statistically significant at *p*<0.05. See supplement for multivariate and spatial statistics.

### Role of the funders

The funders had no role in the study design, data collection, and analysis, decision to publish, or preparation of the manuscript.

## Results

### Demographics and clinical features

Lung samples from 18 COVID-19 patients (6F/12M), median age of 65 years, were analyzed. All patients had confirmation of SARS-CoV-2 infection by a positive RT-PCR result on the naso/oropharyngeal swab and/or lung tissue. Part of this population has been previously presented.^5^^,^^6^ Tables 1 and 2 show patients’ demographics, comorbidities, and clinical characteristics.

**Table 1:** Demographic characteristics of COVID-19 and control patients.

	COVID-19 (n=18)	DAD non-COVID (n=3)	Control (n=6)
**Age in years,** median (range)	65 (33-88)	73 (34-73)	68·5 (51-75)
**Body Mass Index,** median (range)	27·1 (13·1-38·6)	29·3 (18·8-49·5)	25·9 (22-35)
**Sex,** n (%)			
Male	12 (66·7%)	2 (66·7%)	4 (66·6%)
Female	6 (33·3%)	1 (33·3%)	2 (33·3%)
**Race (self-declared),** n (%)			
White	15 (83·3%)	3 (100%)	2 (33·3%)
Afro-descendent	3 (16·7%)	0	4 (66·7%)
**Comorbidities,** n (%)			
SAH	10 (55·6%)	2 (66·7%)	5 (83·3%)
Diabetes mellitus	7 (38·9%)	2 (66·7%)	3 (50%)
Cardiopathy	7 (38·9%)	0	0
COPD	4 (22·2%)	0	0
Obesity	2 (13·3%)	1 (33·3%)	0
Asthma	1 (5·6%)	0	0
Breast cancer	1 (5·6%)	0	0
Chronic renal disease	1 (5·6%)	0	0
Stroke	2 (11·1%)	0	0
No relevant comorbidities	4 (26·7%)	0	0
**Smoking,** n (%)			
Yes	1 (5·6%)	0	1 (16·7%)
Former	8 (44·4%)	0	0

DAD, diffuse alveolar damage; SAH, Systemic Arterial Hypertension; COPD, Chronic Obstructive Pulmonary Disease. The variables presented in this table showed no statistical differences among the groups, except for the self-declared race (Chi-Square Test *p*-value= 0·03). We have not adjusted for race in our data analysis.

**Table 2:** Clinical characteristics of COVID-19 patients.

Initial Symptoms, n (%)	COVID-19 (n=18)
Dyspnea	15 (83·3%)
Fever	14 (77·8%)
Cough	14 (77·8%)
Rhinorrhea	4 (22·2%)
Nausea/Vomiting	4 (22·2%)
Diarrhea	3 (16·7%)
Sore Throat	1 (5·6%)
Myalgia	1 (5·6%)
**Ventilation,** n (%)	
Orotracheal Intubation	18 (100%)
Prone Position	9 (50%)
ECMO	1 (5·9%)
**Lung parameters from 24 h before death,** median (range)	
PEEP (cmH2O), n = 14	10 (6–20)
FiO_2_ (%), n = 14	72·5 (40–100)
PaO_2_/FiO_2_ ratio, n = 12	103·5 (76 – 195)
**Laboratory parameters from 24 h before death**, median (range)	
Leucocytes (1·000/mm^3^), n = 17	10·86 (4·98 – 27·03)
Lymphocytes (1·000/mm^3^), n = 17	0·52 (0·07 – 1·05)
Platelets (1·000/mm^3^), n = 17	176·000 (58·000 – 322·000)
CRP (mg/L), n=14	291·45 (23·6 – 511)
Lactate (mg/dL), n=16	15·5 (9-100)
Troponin (ng/mL), n=11	0·071 (0·01 – 99)
Activated partial thromboplastin time(s), n = 11	1·2 (0·9 – 4·6)
D Dimer (ng/mL), n = 9	2058 (976–89513)
Fibrinogen (mg/dL), n = 5	418 (403–684)
**Time from symptom onset to hospitalization in days**, median (range)	10 (0 – 26)
**Time from symptom onset to death in days**, median (range)	14·5 (3-37)
**Period of hospitalization in days**, median (range)	7·8 (0 – 26)
**Mechanical ventilation length in days**, median (range)	8 (0 – 21)
**Intensive care unit stay in days**, median (range)	7·5 (0 – 25)

ECMO: extracorporeal membrane oxygenation; PEEP, Positive end-expiratory pressure; FiO_2_, Fraction of inspired oxygen ratio; PaO_2_/FiO_2_, Ratio arterial oxygen partial pressure/inspired oxygen fraction ratio; CRP, C-reactive protein.

### Histopathological features in H&E-stained lung sections

**Figure 1:**
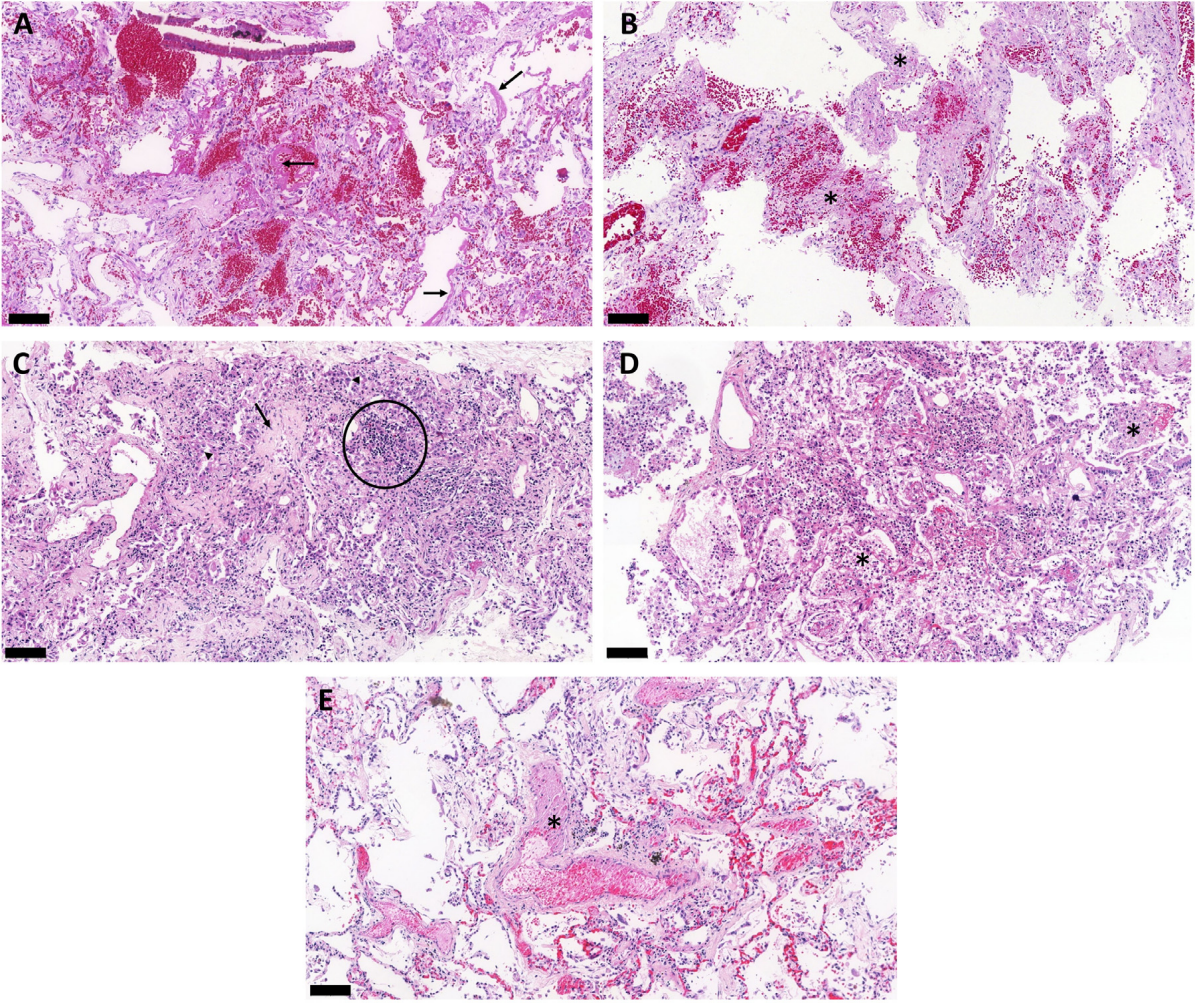
**H&E-stained lung biopsies from ultrasound-guided minimally invasive autopsies confirm typical pulmonary histological findings of fatal cases of COVID-19**. (A) Exudative diffuse alveolar damage with hyaline membranes (arrows) in the alveolar space, alveolar oedema, and congestion. (B) Mixed pattern of diffuse alveolar damage showing the combination of areas of haemorrhage and thickened alveolar septa with loose collagen deposition. Asterisks show the septal thickening. (C) Proliferative phase of diffuse alveolar damage showing thickened alveolar septa with deposition of collagen, lymphocytic infiltrate, and reactive and proliferative type II pneumocytes during alveolar reepithelization. Circled area shows the lymphocytic infiltrate; arrow points to collagen deposition and arrowheads indicate the proliferation of type II pneumocytes. (D) Secondary suppurative bacterial pneumonia characterized by intra alveolar exudation with macrophages and polymorphonuclear infiltrate, indicated by the asterisks. (E) Pulmonary artery with thrombus, indicated by the asterisk. Scale bar = 100 µm.

Lung biopsies of COVID-19 patients were markedly heterogeneous regarding features of DAD, bronchopneumonia, and hemorrhage. All patients had DAD, 16 patients had arteriolar and/or capillary thrombosis and seven had histological signs of bronchopneumonia (Table S4). Non-COVID-19 DAD cases presented features of exudative DAD, and controls had histologically normal lungs. Table 3 provides the percentage of lung involvement by different pathological patterns and histological findings in the post-mortem lung biopsies of the COVID-19 cases. Histological patterns of COVID-19 cases are shown in Figure 1.

**Table 3:** Percentage of lung involvement by different pathological patterns and histological findings in lung post-mortem biopsies of COVID-19 patients.

	Median (range), n=18
**Normal lung (%)**	7·94 (0–35)
**Acute/Exudative DAD (%)**	15·94 (0–93)
**Intermediate DAD (%)**	40·88 (1–99)
**Advanced DAD (%)**	1·88 (0–93)
**Alveolar hemorrhage (%)**	0·69 (0–10)
**Infarct (%)**	0 (0–9)
**Bronchopneumonia (%)**	0 (0–16)
**Arteriolar thrombosis (number)**^a^	0 (0–10)
**Capillary thrombosis (sum)**^b^	0 (0–5)

DAD: Diffuse alveolar damage.

a Number of vessels with thrombosis in each patient. Fourteen out of 18 COVID-19 patients presented arteriolar thrombosis.

b Sum of biopsied lung sites presenting capillary thrombosis in each patient. Eight out of the 18 COVID-19 patients presented capillary thrombosis.

### Immune and structural cell quantification by multiplex immunohistochemistry

#### Quantification of total immune and structural cells: comparison among COVID-19 DAD vs. non-COVID-19 DAD vs. controls

Using raw histomic data from the multiplex images, we compared the total marker density for immune and structural cells among the three study groups. CD8+T cells were increased in COVID-19 cases compared to non-COVID-19 DAD cases, whereas tryptase+ mast cells and Ki67+ cells were increased in COVID-19 related to controls. Macrophages, epithelial, endothelial, and fibroblasts were increased in COVID-19 cases compared to non-COVID-19 DAD and controls. Lung tissue densities of inflammatory and structural cells among COVID-19 and controls are shown in Table 4.

**Table 4:** Total quantification of structural and immune cells in the lung tissue.

Cell Type / Marker	Control	Non-COVID-19 DAD	COVID-19
**Structural Cells**			
Epithelium (cytokeratin)	9·0 (4·8 - 27·4) *	3·2 (2·6 - 5·1) *	54·3 (14·3 - 125·2)
Endothelium (CD31)	9·92 (2·7 - 33·9) *	5·23 (5·1 - 24·1) *	51·8 (19·8 - 79·9)
Fibroblasts (vimentin)	31·5 (7·9 - 55·2) *	10·2 (2 - 18·9) *	59·9 (33·7 - 106·6)
Lymph Vessels (D240)	3·6 (0·5 - 8·4)	1·1 (0·2 - 7·4)	5·1 (0·1 - 13·7)
Smooth muscle (aSMA)	31·4 (22 - 40·2)	23·5 (17·9 - 26·8)	30·6 (22·9 - 67·8)
**Immune Cells**			
Eosinophils (ECP)	0·04 (0·005 - 0·2)	0·02 (0·009 - 0·03)	0·05 (0 - 0·3)
Basophils (pro-BPP)	0·02 (0·006 - 0·06)	0·03 (0·006 - 0·5)	0·11 (0·003 - 0·7)
Neutrophils (MPO)	6·5 (3·4 - 23·5)	10·8 (5·4 - 46)	12·3 (2·9 - 26·9)
B-lymphocytes (CD20)	0·4 (0·1 - 1·9)	0·3 (0·01 - 0·9)	1·1 (0·2 - 2·8)
Th lymphocytes [CD4 (CD3+CD8-)]	0·2 (0·02 - 1·9)	0·001 (0 - 0·2)	0·9 (0·2 - 4·2)
T lymphocytes (CD8)	0·8 (0·01 - 1·8)	0·001 (0 - 0·4) *	2·2 (0·008 - 5·9)
Macrophages (CD68+CD163)	8·9 (2·2 - 23·8) *	20·9 (5·8 - 70·2) *	82·9 (39·6 - 148·4)
Mast cells (Tryptase)	0·5 (0·04 - 0·6) *	0·9 (0·1 - 1·5)	1·7 (0·6 - 3·6)
Langerin DC (CD207)	0·04 (0·01 - 0·4)	0·01 (0 - 0·02)	0·01 (0 - 0·09)
Myeloid DC (CD11c)	5·2 (1·4 - 11·4)	0·9 (0·02 - 6·1)	4·8 (0·2 - 22·9)
**Others**			
Anthracotic Pigment	2·2 (1·6 - 5·2)	2·5 (0·3 - 2·9)	0·9 (0·3 - 19·2)
Collagen I	54·2 (0·2 - 137·5)	5·9 (0·003 - 7·9)	21·3 (1·2 - 78·4)
Proliferation (Ki67)	0 (0 - 0·003) *	0 (0 - 0·002)	2·6 (0 - 9·1)
Red Blood Cells (Glycophorin)	56·2 (7·7 - 107·4)	36·3 (23·7 - 190·9)	21·3 (4·4 - 74·6)
Total Cell Density (Htx nuclei)	87·2 (37·2 - 109·7)	86·36 (63·9 - 135·5)	86·9 (47·3 - 156·6)

Data are presented as fraction of the marker-positive tissue area per lung tissue area (‰). Data are expressed in median (min - max). DC, Dendritic cells; DAD, Diffuse alveolar damage; ns, not significant. * *p*<0·05 compared to the COVID-19 group.

#### Correlations between lung tissue immune and structural cells, lung tissue cytokines, and clinical data from COVID-19 patients

**Figure 2:**
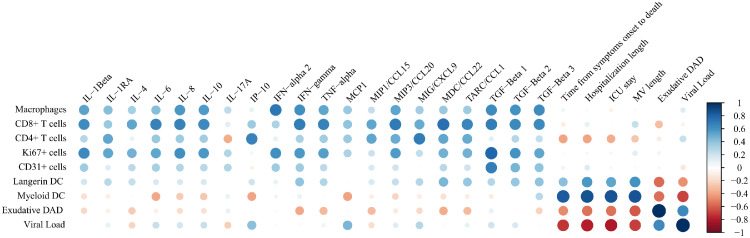
**Correlation plot showing the significant correlations between immune and structural cell quantifications, cytokines, and clinical data of COVID-19.** The specific correlation coefficients and *p*-values are shown in Table S5. DAD, Diffuse Alveolar Damage; DC, Dendritic cells; ICU, Intensive Care Unit; MV, Mechanical Ventilation.

The cytokines individual values of COVID-19 patients are presented in Table S4. Tissue infiltrating macrophages, CD4+ and CD8+ T cells were the immune cells that presented most of the correlations with several cytokines. Interestingly, myeloid dendritic cells (DC) presented strong positive correlations with parameters of the duration of disease whereas being negatively correlated with the percentage of exudative DAD and viral load. See Figure 2 and Table S5.

#### Multiplex-based identification of spatially complex and highly compartmentalized immunopathological and DAD patterns in COVID-19 lung samples

**Figure 3:**
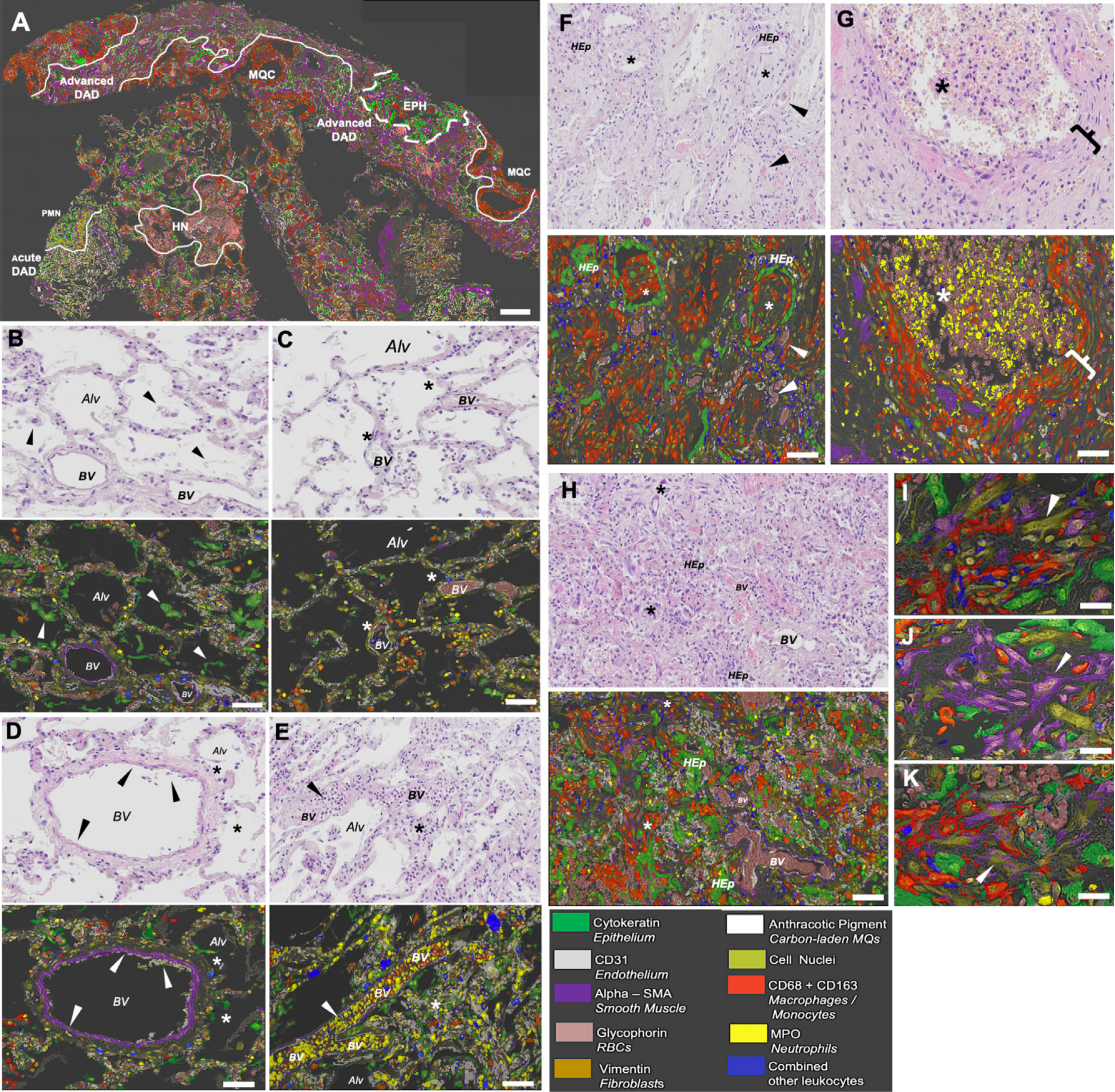
**Multiplex imaging identifies complex patchworks of parallel histopathological microenvironments as a common feature in fatal COVID-19: Exemplification of the histopathological heterogeneity by combined H&E and multiplex IHC imaging in a single COVID-19 lung biopsy.** (A) Low power image from a COVID-19 lung biopsy where the main structural cell markers, neutrophils, and macrophages are highlighted. The other immune cell markers are combined into a blue label to reduce the visual complexity. The marked patchy and compartmentalized histopathological heterogeneity is seen as distinct microenvironments with concomitant exudative DAD areas, localized neutrophilia (PMN), hemorrhage and necrosis (HN), macrophage and fibroblast-rich clusters (MQC), alveolar epithelial hyperplasia (EPH) and fibrotic lung tissue in advanced DAD areas. (B-H) Zoomed-in pairwise images with routine H&E staining (upper panel) and corresponding multiplex IHC image (lower panel). (B) Area with exudative DAD and marked ongoing shedding of the alveolar epithelium (green, arrowheads). (C) Exudative DAD with almost complete denudation of epithelial cells (asterisk) and mild early influx of neutrophils (yellow). (D) Pulmonary blood vessel with focal loss of endothelium. (E) Blood vessel occluded with neutrophils (yellow) and monocytes (red) in an area with alveolar epithelial and vascular disarray. (F) Intermediate DAD with epithelial hyperplasia accompanied by macrophages (red) and fibroblasts (brown; asterisks denote intraluminal fibrosis; arrowheads denote microthrombosis). (G) Neutrophil and hemorrhage space (asterisk) flanked with dense macrophage and fibroblast sheets (bracket). (H) Advanced DAD with epithelial hyperplasia (Hep) and emergence of organized fibroblasts, solitary smooth muscle cells, and myofibroblasts (asterisks). (I-K) High power images exemplifying fibroblasts (I), solitary smooth muscle cells (J), and aSMA^+^, vimentin^+^ double-positive myofibroblast (K) associated with COVID-19 associated advanced DAD. BV= blood vessel, Alv = alveolar space, Hep = Hyperplastic epithelium. Scale bars: A = 0.6 mm; B-H = 70 µm; I-K = 20 µm.

Patchy microenvironments displaying distinct marker combinations could be clearly observed within lung biopsies from the COVID-19 cases. Figure 3A exemplifies the typical spatial heterogeneity of a COVID-19 case, with different sub-millimeter microenvironments within distinct DAD patterns that occur simultaneously within a single biopsy/section. In areas of exudative DAD, there was extensive shedding of alveolar epithelial cells (Figure 3B), and denuded alveolar walls (Figure 3C) along with hyaline membranes. The epithelial shedding was paralleled with vascular alterations like loss of endothelial cells, thrombosis, and vessel occlusion by tethered intravascular neutrophils and monocytes (Figure 3D and E). Alongside areas of exudative DAD, there was also intermediate DAD with dense sheets of fibroblasts, macrophages, patchy hyperplastic epithelium, and alveolar spaces rich in neutrophils, hemorrhage, and cell debris (Figure 3F and G). An advanced DAD area in the same biopsy displayed organized fibroblasts, solitary smooth muscle cells, and vimentin^+^, aSMA^+^ CD31^−^ myofibroblasts (Figure 3H-K). In addition, non-COVID-19 alterations such as clusters of carbon-laden macrophages were also identified (Figure 3A). Figure S1 represents another example of the phenomenon of multiple immunopathological microenvironments occurring simultaneously within a single biopsy. Figure S2 shows the patchy and marked lymphangiogenesis observed in some of the COVID-19 cases.

Proliferation, as measured by Ki67+ cells nuclei, was multifaceted and variably observed in multiple populations of both structural cells (epithelial cells, fibroblasts, lymphatics blood vessel endothelium, and smooth muscle cells) and leukocytes (CD4 T cells, CD20+ B-lymphocytes, macrophages, and neutrophils). Details on proliferating cells are shown in Figure S3.

**Figure 4:**
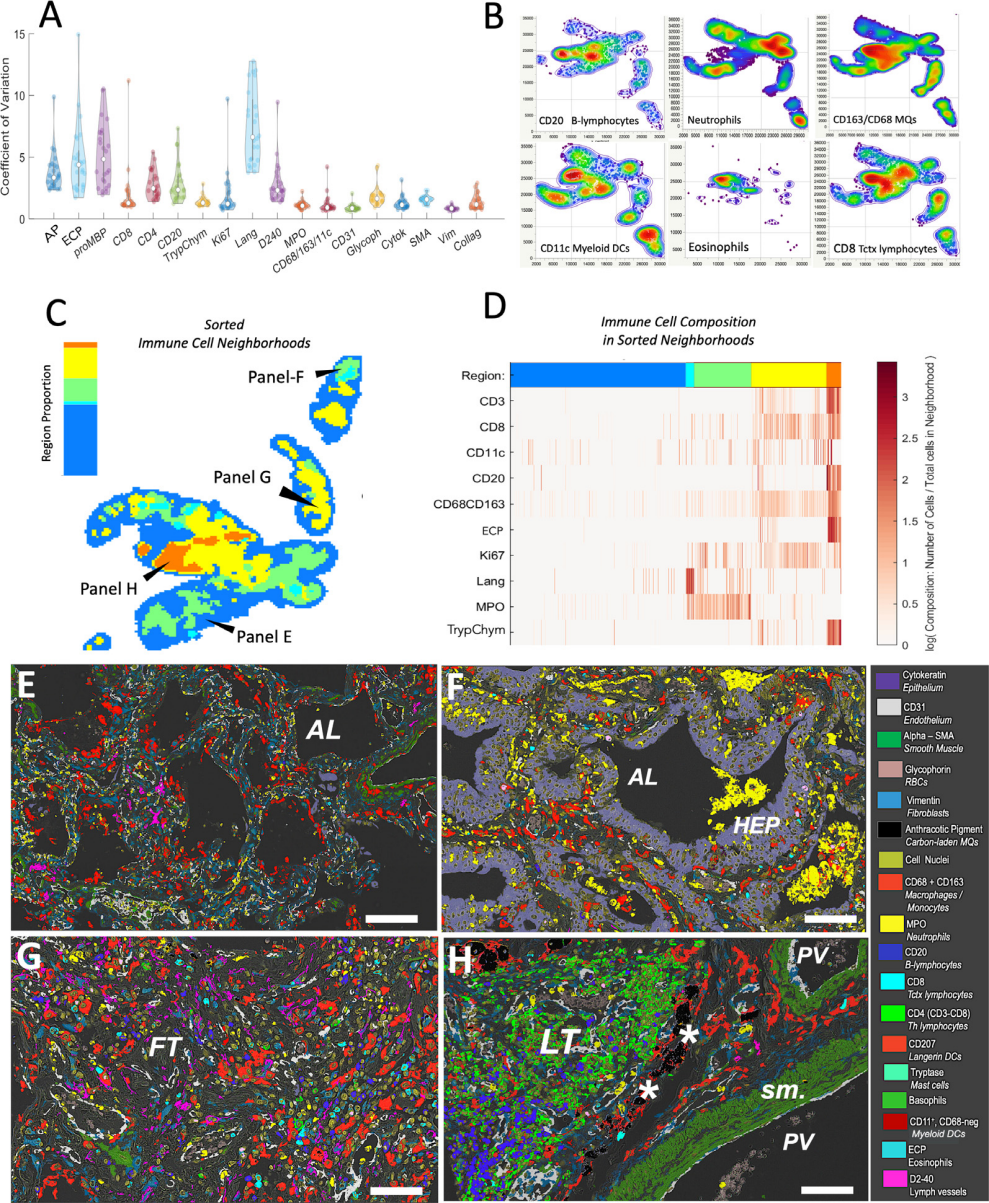
**Formation of heterogeneous and compartmentalized immune cell niches in COVID lungs**. (A) Adjusted coefficient of spatial variation across the IHC multiplex markers. Each dot represents the mean coefficient of variation per marker for the hundreds of analyzed pre-defined 1000 × 1000-pixel lung tissue areas explored per sample. Statistical differences between markers (see *p*-values in the text) were determined by a non-parametric Dunn´s test for all pairs with Bonferroni adjustment. (B) Different spatial distribution patterns among immune cell populations. Panels exemplify color-coded weighted spatial density maps for B-lymphocytes, neutrophils, monocytes/macrophages, myeloid DCs, eosinophils, and CD8 T-lymphocytes. The density plots are based on cell x,y coordinates within the same tissue section and display density gradients from low (blue) to high (red). (C-D) Example of AI-based non-supervised identification of spatial immune cell patterns in a single lung section. The analysis was performed by the CytoMAP platform^7^ using marker identity and x,y coordinates for individual immune cells. The spatial distribution of regions with identified and color-coded immune cell neighborhoods is shown in C, whereas the corresponding relative immune cell compositions are shown in panel D. (E-H) Corresponding multiplex micrograph images from AI-identified tissue regions are outlined in panel C. (E) Intermediate DAD; (F) marked epithelial hyperplasia and neutrophil infiltration; (G) advanced DAD, (H) region with pigmented macrophages and lymphoid tissue. AP = anthracotic pigment, ECP = the eosinophil marker eosinophil cationic protein, Trypt/Chym = Mast cell markers, Lang = langerin/CD207 DCs, MPO = neutrophil marker, vim = the fibroblast marker vimentin, Glycoph = red blood cell marker, Cytoker = epithelial marker, SMA smooth muscle actin, vim = the fibroblast marker vimentin, Collag = Collagen 1, AL=alveolar lumen, FT= fibrotic tissue, LT = lymphoid tissue. Scale bars: E and F = 65 µm; G and H = 50 µm.

The digital histomic data from the multiplex digital images were used to further explore the spatial heterogeneity. We applied a density-based grid approach, exploring marker content in hundreds of 1000 × 1000 pixel size areas within each biopsy of COVID-19 patients to demonstrate that immune cells had more variable distribution than structural cell markers. Langerin DC, eosinophils, and basophils displaying the highest spatial variability (*p*<0·001), followed by anthracotic pigment (*p*=0·007), CD20+ B-cells (*p*=0·009) and, CD4+ T-cells (*p*=0·03) (Figure 4A). Interestingly, lymph vessels displayed high spatial heterogeneity compared to the other structural markers (*p*<0·001; Figure 4A). Analysis of the density distribution maps in the different biopsies revealed the common phenomenon of immune cells population having distinct distribution patterns (Figure 4B). Next, we used an AI-based point pattern (x,y coordinate)-based approach for unsupervised identification of immune cell niches^7^ (Figure 4C-D). As an example, in a single biopsy, five spatially heterogeneous immune cell neighborhood areas were identified (C), each with a distinct relative composition of immune cells (D). Superimposing the AI-identified immune cell niches (Figure 4C-D) with the multiplex images revealed that the immune cell patterns were generally linked to distinct areas of the various patterns of DAD or other features such as clusters of anthracosis and/or areas with ectopic lymphoid tissue (Figure 4E-H).

#### Immune and structural cell profiling of DAD patterns

**Figure 5:**
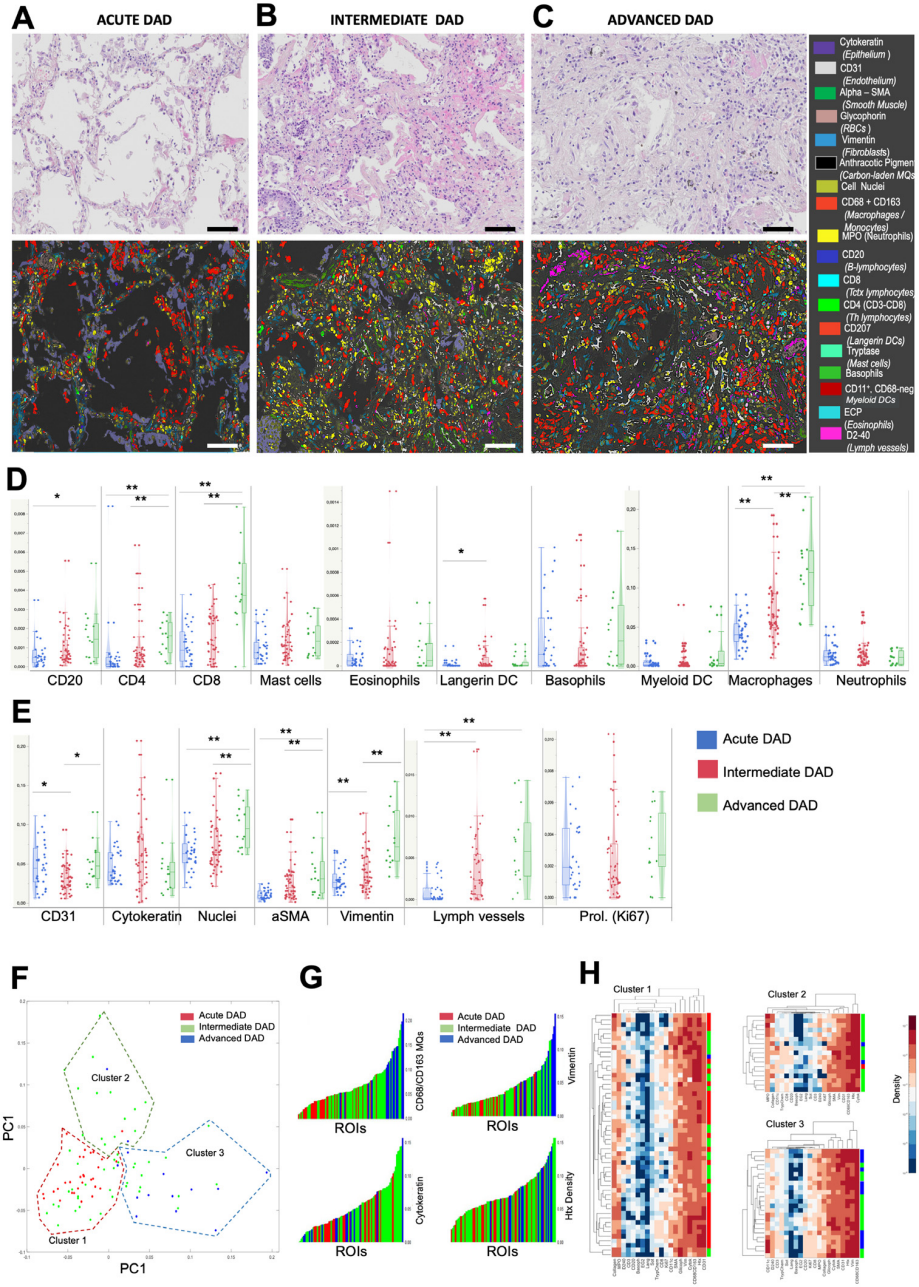
**Decoding of cell marker compositions during DAD progression**. (A-C) Zoomed-in micrographs of diffuse alveolar damage (DAD) patterns, as viewed by traditional H&E staining and corresponding multiplex immunohistochemistry. Panels A-C show paired H&E and multiplex images and typical patterns during exudative, intermediate, and advanced DAD, respectively. (D-E) Quantitative data on the density of immune cells (D) and structural cell markers (E) across the DAD patterns. The data are from marker density analysis in 95 multiplex-stained tissue regions of interest (ROIs) that were selected from H&E-stained sections with the criteria of having a uniform and distinct DAD histopathology. Statistical comparisons were determined by a non-parametric Kruskal–Wallis test, followed by Bonferroni post-hoc test. (F) Multivariate analysis of individual DAD region marker content and identification of 3 clusters of marker constellations by principal component analysis (PCA) and unsupervised K-mean clustering (Clusters 1-3). Individual ROIs within the PCA-defined clusters are color-coded according to previously H&E-confirmed DAD patterns. (G) Individual ROIs sorted for increasing abundance of the 4 markers that had the most statistical influence on initial cluster identification, again individual ROIs are color-coded according to DAD category. (H) Cell plots with relative marker densities across the identified clusters. Each horizontal line represents one DAD region; with its DAD category color-coding to the right. *p<0·05 and **p<0·01.

The complex patchwork of concomitant DAD stages provided a rationale to “filter out” and selectively explore microenvironments with uniform DAD patterns. Accordingly, among the COVID-19 cases ninety-five histologically homogenous tissue areas of exudative, intermediate, or advanced DAD were identified for quantitative decoding of changes in immune cell profiles and structural damages across the DAD patterns (Figure S4 and Figure 5A-C). Among the immune cells, macrophages, CD20+B cells, and CD4 and CD8+T lymphocytes gradually increased during the progression from exudative to advanced DAD (Figure 5D). Macrophages in more acute DAD patterns generally had a more lysozyme (M1)-tilted phenotype, whereas macrophages in more advanced DAD stages were more CD206 (M2)-skewed. Although a high degree of marker heterogeneity was observed, interstitial macrophages generally had a more CD206-skewed type than corresponding luminal cells (Figure S5).

Langerin DC increased from exudative to intermediate DAD (Figure 5D). Alpha-smooth muscle actin, vimentin, and lymphatic vessels displayed a robust increase from exudative to advanced DAD (Figure 5E). CD31+ blood vessels peaked at intermediate DAD, whereas cytokeratin showed a non-significant trend towards an increase at intermediate DAD (Figure 5E).

Total cellularity increased during the DAD progression whereas cell proliferation was constant during the three DAD patterns (Figure 5E). The complete set of marker data from each of the 95 DAD ROIs were subjected to multivariate analysis. Three clusters were identified by PCA and unsupervised K-mean clustering (Figure 5F). Decoding of the ROIs, DAD status revealed that the unsupervised cluster identification largely corresponds to the manually defined DAD patterns. Ninety-five percent of the exudative DAD ROIs were found in cluster 1, 91% of the advanced ROI in cluster 3, whereas intermediate DAD ROIs dominated in cluster 2 (Figure 5F). Loading information analysis identified macrophages and vimentin as the most influential markers belonging to cluster 3 and high cytokeratin as a strong predictor of falling within cluster 2 (Figure S6). This behavior was further revealed by looking at the color-coding of all DAD ROIs and sorting them for increasing CD68, vimentin, cytokeratin, and total cell density (Figure 5G). The relative density for all markers and DAD ROIs within the PCA-identified clusters are presented in Figure 5H.

### PCR-based viral load assessment of SARS-CoV-2 and non-viral co-infections

The viral load of SARS-CoV-2 in the lung tissues had a median of 1,380 virus copies per 100,000 cells (range 13 – 88,985 virus copies per 100,000 cells). See Table S5 for correlations.

All cases presented co-infections by at least one microorganism. The most frequent were the gram-negative bacteria such as *Escherichia coli* and *Shigella flexneri. Candida parapsilosis* was present in 8 cases. Co-infections in all COVID-19 patients are presented in Table S4.

SARS-CoV2 mRNA density was low with only a few scattered SARS-CoV2 mRNA dots (Figure S7A) in most of the cases. A few cases with exudative DAD regions had more detectable viral mRNA (Figure S7B, C, E, and G). The tissue fraction area for SARS-CoV-2 staining was 9·6E-3% (range 0·5 – 117 E-3).

Bacterial levels, assessed as ISH detection of 16SrRNA, were absent or low, exemplified in Figure S7D and I. The tissue area fraction of 16SrRNA was 8.4 E-4 % (range 0 – 4·1 E-3).

Tissue ROIs were selected from sections with patchy and variable degrees of SARS-CoV-2 or bacteria. Corresponding tissue areas in neighboring multiplex IHC sections showed that regions with higher SARS-CoV-2 levels had more lymphocytes than the few bacterial regions, which were characterized by abundant neutrophils (Figure S7K and L).

### Transcriptomics

RNA extraction obtained on average approximately 12 million 151-bp paired-end reads with high quality (average phred score per read ≥ 30) for each sample. Only those reads that aligned to the human genome were used in downstream analyses (Table S6).

Five cases had a predominantly exudative DAD pattern, and seven cases had intermediate/advanced DAD. PCA showed that intermediate/advanced DAD samples grouped together, while exudative DAD samples were scattered (Figure S8). The differential expression analysis between each of these two groups and the control cases revealed 261 and 244 DEGs in the exudative and intermediate/advanced DAD tissues respectively; 61 DEGs were shared between them (Table 5, Table S7, Figure S9, Table S8).

**Table 5:** Number of DEGs in exudative DAD and mixed/advanced DAD tissues when comparing each one with control lung tissues and the number of DEGs shared between the two DAD patterns.

	Acute DAD	Mixed/Advanced DAD	Shared
**Up-regulated**	143	67	45
**Down-regulated**	57	116	16

DAD, diffuse alveolar damage.

We determined GO terms and KEGG pathways enriched in each comparison (Tables S9 - S11). Patients with exudative DAD had up-regulated genes related to oxidative phosphorylation, anti-microbial responses, blood coagulation, megakaryocytes differentiation/regulation, and platelet degranulation/activation. Patients with intermediate/advanced DAD presented up-regulated genes related to immunoglobulins and extracellular matrix organization. The shared up-regulated DEGs between both histological patterns are involved in innate and adaptive immune responses, probably a shared response to SARS-CoV-2 infection.

We selected six DEGs (three DEGs in each histological pattern) for validation by real-time PCR. They were chosen based on their high log fold change values and functional annotation of interest (Figure S10 and S11). There were no significant differences between controls and the three DEG genes overexpressed in exudative DAD, but all the three DEG overexpressed in intermediate DAD were significantly higher by RT-PCR analysis (Table 6).

**Table 6:** Relative Gene Expression of six candidate genes between control and exudative or intermediate/advanced DAD pattern to validate the differential expression assessed by Real-Time PCR in lung tissue.

Gene	Control x Exudative DAD	*p*-value
PI3	1·12 (0·48 – 1·96) x 0·55 (0·08 – 420·96)	ns
PGLYRP1	1·08 (0·61 – 1·56) x 2·33 (0·27 – 92·81)	ns
GP9	0·98 (0·85 – 1·21) x 1·11 (0·67 – 1·58)	ns
	Control x Intermediate/Advanced DAD	
COL3A1	1·31 (0·32 – 2·83) x 15·78 (5·45 – 83·35)	0·006
IGLV3-19	0·7 (0·53 – 3·87) x 31·03 (4·42 – 834·6)	0·006
IGHV1-58	1·24 (0·21 – 4·28) x 36·18 (6·89 – 528·06)	0·006

Data expressed in median (min-max). ns, not significant; PI3, Peptidase inhibitor 3; PGLYRP1, Peptidoglycan recognition protein 1; GP9, Glycoprotein IX platelet; COL3A1, Collagen type III alpha 1 chain; IGLV3-19, Immunoglobulin lambda variable 3-19; IGHV1-58, Immunoglobulin heavy variable 1-58.

## Discussion

In this study, we provided a holistic and yet spatially decoded comprehension of lung involvement in fatal COVID-19, a disease with a highly heterogeneous individual pathology with coexisting different DAD patterns. We showed that COVID-19 lung disease was characterized by an intense proliferative lung response, alternating different spatial and temporal areas of exudative DAD, rich in macrophages/neutrophils, a higher virus load, and many DEGs related to acute responses and thrombotic phenomena. These coexisted with different areas of intermediate/advanced DAD, with mounting macrophagic, lymphocytic, and DC immune responses, epithelial hyperplasia, (lymph)angiogenesis and structural remodeling, a lower viral load and DEGs related to immunoglobulin/extracellular matrix production. These phenomena were linked with a cytokine-rich milieu and the presence of enteric bacteria and *Candida sp* in lung tissue. Few studies performed a detailed analysis of the spatial immunological and structural components within the heterogeneous cyclic mosaic of DAD lesions in COVID-19. It is believed that understanding such heterogeneity is important to be recognized in the management of patients and in the development of novel treatment strategies.^11^

Our observation of a correlation between SARS-CoV-2 viral load and exudative, but not intermediate/advanced DAD regions, supports the ‘hit and run” strategy, where the time course of virus tissue-damaging infection-replication-propagation sequence cycle is short.^12^ We postulate that virus spread within the lungs causes different cycles of local patchy foci of acute damage. A mounting cellular and humoral immune responses and tissue organization take place, regardless of the viral presence, successively, until, in severe cases, the lung is fully involved. Dorward et al.^13^ has previously indicated the dissociation of the topologically associated lung pathology with the virus presence. Therefore, understanding the spatially different lesions in the COVID-19 lungs is pivotal to better envisioning the disease complexity of concomitant areas of lung damage and repair.

When compared to controls, patients with COVID-19 associated DAD presented increased proliferating cells, reflected by the increase of the total density of the immune cells CD8+T cells, macrophages, and mast cells. The density of structural cells such as epithelial cells, endothelium, and fibroblasts was also increased (Table 4). However, such total tissue quantification does not envision the heterogeneity and complexity of cell arrangements present within each lung biopsy (Figure 3) and may reflect the divergent results in the literature, especially in the quantification of lymphocytes.^14^

Whereas in exudative DAD the phenomena of denudation of epithelial and endothelial cells prevail, the proliferation of different structural cells and a mounting cellular immune response occurs in the intermediate/advanced phases, coupled to a lower viral load. Our data provide novel information showing that the T and B-lymphocyte density increased in intermediate/advanced DAD and it was associated with a peak of DC in the intermediate phase that correlated negatively with the virus load. Autopsy reports describe variable amounts of lymphocytes in lung tissue.^6^ Previous data show that there is an increase in activated CD4+/CD8+ T lymphocytes in the lungs of critical COVID-19 patients.^15^ Lymphocytic response in COVID-19 has been subjected to intense research, and data point to suboptimal, excessive or otherwise inappropriate T-cell responses associated with severe disease, with high heterogeneity in gene expression of CD8 T cells in critical patients.^16^ Our data suggest that such conflicting results might reflect the tissue heterogeneity, results depending on the type of lung microenvironment material being sampled. In addition to antiviral cytokines, CD8+T cells correlated with all the three isoforms of TGF-beta, indicating a direction towards the tissue-resident memory phenotype.^17^ Memory T cells might have an immune-protective capacity, but also might mediate tissue pathology during the disease course leading to non-resolution of lung inflammation and fibrosis, especially in aged hosts.^18^

Macrophages are key cells in the COVID-19 immunopathology.^19^ Single-cell analysis identified the inflammatory macrophage phenotype in the lungs of patients with critical COVID-19.^20^ Our data show that *in situ* proliferation significantly contributes to the sustained increased amount of these cells in the lungs of critical cases, forming on some occasions, pseudopalisades of cells. Macrophages correlated with many cytokines with antiviral properties such as interferons, supporting evidence of their role in antiviral defenses. In addition, Wendisch et al.^21^ identified a profibrotic transcriptional phenotype in macrophages during COVID-19 ARDS. Our observation of a foremost lysozyme macrophage phenotype in acute DAD and a more CD206-skewed profile in advanced DAD is in accordance with “M1 macrophages” being pro-inflammatory and responding to e.g., IFN-gamma and M2´s suggested role in repair, and resolving inflammation.^19^ However, considering the vast plasticity of macrophages, further and quantitative studies are warranted to get a more complete picture of the complex role of macrophages as key immune and repair regulators in COVID-affected lungs.

Both epithelial cells and DC peaked in the intermediate DAD. In animals, alveolar DC tended to increase later after lung injury, with alveolar epithelial cells orchestrating DC function.^22^^,^^23^

Little is known about the dynamics of DC in human DAD but interestingly the present peak of DC in intermediate DAD coincides with the expansion of B- and T-lymphocytes, hyperplastic alveolar epithelium, and local lymphangiogenesis. Taken together, this suggests that progression of DAD is associated with the formation of a novel axis of reprogrammed alveolar epithelium -DC - B/T lymphocyte – lymphatics, a phenomenon that may provide a structural basis for increased expansion of adaptive immune responses to other organs.

Few studies have isolated bacteria and fungi in the distal lung tissue of patients who died of COVID-19. Low quantities of bacterial DNA were present in all cases, identified as enteric, gram-negative bacteria, a result similar to Fan et al.^24^ Gaibani et al.^25^ showed the predominance of gram-negative bacteria with multidrug resistance phenotypes in the lung microbiome of COVID-19 patients. In our study, five patients had *Staphylococcus sp* isolated from tracheal secretions, but no enteric bacteria, suggesting different agents in more distal parts of the lung. Secondary fungal infections have been reported in severe cases of COVID-19.^26^ The finding of *C. parapsilosis* is in accordance with the emergence of this yeast, worldwide and in our region, as a nosocomial pathogen.^27^ It has been postulated that SARS-CoV-2 affects the gastrointestinal tract, disrupting mucosal immunity and altering the microbiota, which may be associated with secondary infections.^28^ It remains challenging to understand how the presence of bacteria and fungi, by causing a local influx of neutrophils as observed in this study, may affect (e.g., with increased NET formation^29^) DAD evolution.

The frequent thrombotic changes were accompanied by endothelial cells abnormalities, angiogenesis with proliferating CD31+ cells, and disordered lymphangiogenesis. The increase in lymphatic vessels in the intermediate DAD may contribute not only to a systemic spread of immune responses but also to remove the excess of interstitial fluid that is a clinically significant threat in severe COVID-19.

Data on enriched pathways gave support to the spatially decoded analysis. Exudative DAD patterns presented enriched pathways related to viral, microbes, and humoral immune responses, coagulation, regulation of blood circulation, platelet degranulation, and megakaryocyte differentiation. On the other hand, the intermediate/advanced DAD presented enriched pathways related to the humoral and adaptive immune response, lymphocyte activation, complement activation, response to bacteria, and extracellular matrix organization. These results are in accordance with two previous studies that showed that patients with higher viral load, initial lung damage, and fewer infiltrating cells presented transcriptomic profiles related to interferon-stimulated genes. Whereas patients with lower viral loads and more severe lung lesions had more macrophages, CD8+T lymphocytes, and enriched pathways for COL1A1 and other markers of pulmonary fibrosis.^30^^,^^31^

Further, our data showed that multiple genetic pathways leading to pulmonary thrombosis are activated at the early phases of the critical disease. The ongoing and not resolved discussion on when/which patient to introduce therapeutic heparin^32^^,^^33^ pointed out that it might be useful for non-critical cases in terms of survival to hospital discharge with reduced use of organ support. Our data support the concept that intervention in the non-critical phase might prevent these phenomena in patients developing severe disease.

This study has several limitations. We studied cases collected in the early periods of the pandemics in Brazil, and therefore cases lack prolonged periods of invasive ventilation. An advantage of this timing is that the longer the hospitalization, the more non-COVID-19 related factors might influence lung disease. We did not have larger numbers of frozen control tissue to perform all comparative analyses such as cytokines assessment and RT-PCR. The lack of difference between DEG in controls vs exudative DAD might be explained by these low numbers, and by the activation of acute inflammatory pathways in the terminal phase of controls. In addition, we had a limited number of non-COVID-19 DAD cases, not allowing similar decoded analyses. We, therefore, were not able to identify pathological features unique to COVID-19 and discriminate between COVID-19 and non-COVID-19 ARDS. The limited number of controls also did not allow us to adjust for race in our data analysis, however; previous published data indicated that it is difficult to disassociate race from socioeconomic factors in several aspects of COVID-19 in Brazil.^34^ We worked with postmortem biopsies of MIAs that do not provide a full spectrum of lung involvement, such as larger airways or arteries. However, we were able to identify and decode the immunopathology of all main types of histological DAD. Postmortem biopsies, collected at relatively short periods when compared to the conventional autopsy, allowed us to perform a series of different techniques in lung tissue, probably because of a better/more rapid fixation.

In summary, we provide here a holistic approach to the spatial/temporal heterogeneous immunopathology of lung involvement in critical cases of COVID-19. By dissecting the different immune and lung structural arrangements within the three pathologically identifiable DAD patterns, we have shown that complex immunological microenvironments with different lung responses may coexist in the same patient, associated with the presence of secondary infections, thrombotic phenomena, and a cytokine rich milieu, driven by different gene expressions. Our data complement and enrich the understanding of dissociated tissue studies, such as single-cell RNAseq analyses. Any description of COVID-19-related pathophysiological processes and putative novel treatments with highly specific targets might consider this enormous and challenging complexity.

## Contributors

JE: conceptualization, supervision, funding acquisition, project administration, writing -original draft, writing -review and editing. NSXC: formal analysis, methodology, writing - original draft, project administration. JJ: visualization, formal analysis, methodology, investigation. OC: formal analysis and software, methodology. KCD formal analysis, methodology, writing - original draft. CMC: investigation, formal analysis. PS: investigation, formal analysis. CL: investigation, formal analysis. MA: investigation, formal analysis. SCFSL: methodology, formal analysis. AMJr: methodology, formal analysis. LA: formal analysis, methodology, writing – original draft. CSF: formal analysis, methodology. ANDN: formal analysis, methodology, writing – review. RAAM: formal analysis, methodology. JRRP: methodology, supervision. MSGG: methodology, formal analysis. RVP: formal analysis, methodology. JSM: formal analysis, methodology, software, writing- original draft. JCS: formal analysis, software, supervision, writing, review and editing. EPO: methodology, formal analysis. JTF: methodology, formal analysis. CS: investigation, project administration. JMO: data interpretation and manuscript drafting. MAS: data interpretation and manuscript drafting. LFFS: funding acquisition, investigation, project administration, supervision, writing – original draft, and writing – review & editing. PHNS; conceptualization, data curation, formal analysis, supervision, funding acquisition, and writing – review & editing. MD: conceptualization, data curation, formal analysis, funding acquisition, supervision, and writing – review & editing. TM: conceptualization, data curation, formal analysis, validation, visualization, supervision, writing – original draft, and writing – review & editing. Jonas Erjefalt and Thais Mauad have verified the underlying data. All the authors confirm that they had full access to all data in the study and take responsibility to submit for publication.

## Data sharing statement

Data on patients’ data, laboratorial tests and lung cytokine analysis will be available upon request to tmauad@usp.br.

Patient data on histological parameters and marker tissue densities are available upon request to jonas.erjefalt@med.lu.se.

Data on transcriptome reads have been deposited in NCBI's Gene Expression Omnibus and are accessible through GEO Series accession number GSE205099 (https://www.ncbi.nlm.nih.gov/geo/query/acc.cgi?acc=GSE205099).

Pathogens identified and sequenced in the lung tissue were submitted to the Genbank (https://submit.ncbi.nlm.nih.gov/) for public sharing.

## Declaration of interests

J.E. is the founder of Medetect AB, Lund, Sweden. J.J., C.S. and C.L. are employees at Medetect AB.

M.A.S. and J.O. are employees and shareholders at Regeneron Pharmaceuticals.

All other co-authors have no conflict of interest to declare with the subject of the manuscript.

## Appendix Supplementary materials

Image, application 1

Image, application 2

Image, application 3

Image, application 4

Image, application 5

Image, application 6

Image, application 7

Image, application 8

Image, video 9
