# Supplementary material for: Diffuse alveolar damage patterns reflect the immunological and molecular heterogeneity in fatal COVID-19
Source: eBioMedicine. 2022 Aug 24;83:104229. doi: 10.1016/j.ebiom.2022.104229 (PMC9398470; doi:10.1016/j.ebiom.2022.104229)
Supplement: Supplementary file 1 [file mmc1.docx]

**Supplementary appendix**

**Diffuse Alveolar Damage Patterns Reflect the Immunological and Molecular Heterogeneity in Fatal COVID-19**

Jonas S Erjefält ^1,2^, Natália de Souza Xavier Costa ^3^, Jimmie Jönsson ^4^, Olga Cozzolino^1^, Katia Cristina Dantas ^3^, Carl-Magnus Clausson^1^, Premkumar Siddhuraj ^1^, Caroline Lindö ^4^, Manar Alyamani ^1^, Suzete Cleusa Ferreira Spina Lombardi ^5,6^, Alfredo Mendroni Júnior ^5,6^, Leila Antonangelo ^7,8^, Caroline Silvério Faria ^7^, Amaro Nunes Duarte Neto ^3^, Renata Aparecida de Almeida Monteiro ^3^, João Renato Rebello Pinho ^7,9^, Michele Soares Gomes-Gouvêa ^10^, Roberta Verciano Pereira ^7^, Jhonatas Sirino Monteiro ^11^, João Carlos Setubal ^11^, Ellen Pierre de Oliveira ^12^, Jair Theodoro Filho^3^, Caroline Sanden ^4^, Jamie M Orengo ^13^, Matthew A Sleeman ^13^, Luiz Fernando Ferraz da Silva ^3,14^, Paulo Hilário Nascimento Saldiva ^3^, Marisa Dolhnikoff ^3^, Thais Mauad^3.^

^1^ Unit of Airway inflammation, Department of Experimental Medicine Sciences, Lund University, Sweden

^2^ Department of Allergology and Respiratory Medicine, Lund University

^3^ Departamento de Patologia, Faculdade de Medicina da Universidade de São Paulo, São Paulo, Brazil.

^4^Medetect AB, Lund, Sweden

^5^ Divisão de Pesquisa & Medicina Transfusional, Fundação Pró-Sangue Hemocentro de São Paulo, São Paulo, Brazil

^6^ Laboratório Investigação Médica em Patogênese e Terapia dirigida em Onco-Imuno-Hematologia (LIM-31), Departamento de Hematologia, Hospital das Clínicas HCFMUSP, Faculdade de Medicina, Universidade de São Paulo, São Paulo, Brazil

^7^ Laboratório de Investigação Médica (LIM03), Hospital das Clínicas HCFMUSP, Faculdade de Medicina, Universidade de São Paulo, São Paulo, Brazil

^8^ Divisão de Patologia Clínica – Departamento de Patologia, Hospital das Clínicas HCFMUSP, Faculdade de Medicina, Universidade de São Paulo, São Paulo, Brazil

^9^ Hospital Israelita Albert Einstein, São Paulo, Brazil.

^10^Departamento de Gastroenterologia (LIM-07), Faculdade de Medicina da Universidade de São Paulo, São Paulo, Brazil.

^11^ Departamento de Bioquímica, Instituto de Química Universidade de São Paulo, São Paulo, Brazil.

^12^ Departamento de Cardiopneumologia, Instituto do Coração, Faculdade de Medicina da Universidade de São Paulo, São Paulo, Brazil.

^13^ Regeneron Pharmaceuticals, Tarrytown, New York, USA

^14^ Serviço de Verificação de Óbitos da Capital, Universidade de São Paulo, São Paulo, Brazil.

**Supplement**

**Contents**

Supplementary Methods 03

Supplementary Results - Figures 08

References 18

**Supplementary Methods**

**Minimally Invasive Autopsy**

We included 18 adult patients that died between March and April 2020 due to COVID-19. During this period, there were 842 admissions due to COVID-19 in the hospitals linked to the Medical School, with 301 deaths. No vaccines were available at the time.

As controls for the histopathological analyses, we selected postmortem biopsies of three cases with non-viral acute diffuse alveolar damage and six cases of acute cardiovascular deaths with normal lung histology.

The autopsies were performed after written informed consent by the next-of-kin. Minimally Invasive Autopsy- Ultrasound guided (MIA-US) protocol was described previously by Duarte-Neto et al.^1^ and was adapted for COVID-19 for safety reasons ^2^. The post-mortem interval ranged from 3 hours to 23 hours (mean of 15.3 hours). Lung tissue was sampled from eight regions, in a combination of upper and lower chest (lateral and medial, 4 sites) and right and left lungs (2 sides). In each sampling site, six samples were collected. Immediately after sampling, pulmonary samples either were immersed in 10% formalin solution for 24 h or immediately snap frozen at -80^o^ C.  For the multiplex immunohistochemistry, 1 to 2 representative formalin-fixed paraffin-embedded (FFPE) biopsies (3-6 samples per slide) with a DAD picture and no histological signs of secondary bacterial/fungus infection were selected. For the microbiological analyses, 1 to 2 FFPE biopsies with histological signs of secondary infection were selected.

**Multiplex Immunohistochemistry**

A platform for multiplex staining (*Additive Multiplex Labeling Cytochemistry, AMLC Platform, Medetect AB, Sweden*) using the principle of cyclic immunohistochemistry was used for simultaneous visualization of multiple leukocytes and structural cell populations within single lung sections from all COVID-19 patients and control individuals. In brief, cycles of chromogenic and/or fluorescence-based immunohistochemical staining were performed in an automated histochemistry robot. Between each staining round there was a blocking and/or antibody elution process and the generated staining pattern was digitalized by microscope-based whole slide scanning. The accumulated series of marker-specific high-resolution digital images were processed by purpose-built software (*Cell Community Viewer, CCV, Medetect AB, Lund, Sweden*) in order to segment out and assemble the marker-specific data objects and generate high-resolution multiplex images. A separate tissue detection algorithm was used to filter out non-tissue backgrounds (such as alveolar air spaces and the lumen of vessels and bronchioles). The “histomic” raw data, such as the intensity, shape index, and localization (x,y coordinates) for all marker-positive objects were automatically exported to an SQL database for subsequent statistical analysis and visual inspection. Importantly, as a method control, the staining for each antigen (i.e., immunohistochemistry [IHC] cell marker) within the multiplex series was also validated and compared to a parallel corresponding “conventional” single staining. Data are presented as the tissue fraction of marker positivity (i.e., marker-positive pixels per total tissue pixels). The marker list with the primary detection antibodies is presented in Table S1. An example of the type of images and exploration is briefly shown in Video 1 ([*https://drive.google.com/file/d/1yYf009ZZ4iWT_q4CsJY1UST9wEo6Q5dw/view?usp=sharing*](https://drive.google.com/file/d/1yYf009ZZ4iWT_q4CsJY1UST9wEo6Q5dw/view?usp=sharing)*).*

**Immunofluorescence double staining for identification of M1 (lysozyme) and M2 (CD206) macrophages**

For antigen retrieval, the slides were baked at 60 °C for 30 min and pretreated with low pH heat-induced epitope retrieval (HIER) by a pH6 target retrieval solution (#DM829; Dako, Glostrup, Denmark) in a DAKO PT Link HIER machine (PT-link 200; Dako, Glostrup, Denmark). Pretreated slides were rinsed thoroughly in wash buffer (#DM831; Dako, Glostrup, Denmark) and blocked with the serum-free solution (#X0909; Dako, Glostrup, Denmark) for 10 min. Lysozyme-positive macrophages were visualized using a rabbit anti-lysozyme primary antibody (#ab223503, clone: SP329; isotype: IgG; dilution 1:150; Abcam, UK). Following one hour of incubation of the primary antibody, a HRP conjugated anti-Rabbit IgG secondary antibody (ImmPRESS polymer Vector Laboratories, Inc.; Burlingame, CA, USA) was added for 30 minutes and the fluorescence signal in the HRP sites were amplified using the tyramide signal amplification system (#11070 AF 488nm; AAT Bioquest, USA) for 10 minutes. To avoid any potential cross signaling with other future primary antibodies the slides were subjected to an antibody elution step using 2-mercaptoethanol, SDS buffer protocol (2-ME/SDS). Next, macrophage mannose receptor 1 (CD206) was visualized using rabbit anti-MRC1primary antibody (#HPA004114, anti-CD206, polyclonal; dilution 1:50; Prestige Antibodies Powered by Atlas Antibodies, Sigma Aldrich, UK). The primary antibody for CD206 was conjugated using the same secondary antibody which was employed for lysozyme staining and the immunereactive sites were amplified using the tyramide signal amplification system (#11075 AF 546nm(Cy3); AAT Bioquest, USA) for 10 minutes. After the double immune-fluorescence staining for lysozyme (FITC channel) & macrophage mannose receptor 1 (Cy3 channel) was completed, tissue sections were nuclear counterstained using Hoechst solution (#33342, Thermo Scientific, USA) for 10 minutes. Results are presented in a descriptive manner.

**Table S1.** Antibodies used in the multiplex immunohistochemistry.

| **Antigen** | **Cell type** | **Format/Clone** | **Vendor** | **Dilution 1:x** | **Concentration µg/ml** |
| --- | --- | --- | --- | --- | --- |
| CD11C | Myeloid dendritic cells (CD11C+CD68-CD163-) | Mouse IgG2a, clone 5D11 | Leica Biosystems, Buffalo Grove, Illinois, USA | 50 | 0·60 |
| CD163 | Macrophages | Mouse IgG1, clone 10D6 | Leica Biosystems, Buffalo Grove, Illinois, USA | 80 | 6·13 |
| CD20 | B lymphocytes | Mouse IgG2a, clone L26 | Leica Biosystems, Buffalo Grove, Illinois, USA | 500 | 1·90 |
| CD3 | T helper T lymphocytes (CD8-CD3+) | Mouse IgG1, clone F7·2·38 | Agilent (Dako), Santa Clara, California, USA | 50 | 2·76 |
| CD31 | Blood vessels | Mouse IgG1, clone JC70A | Agilent (Dako), Santa Clara, California, USA | 75 | 2·73 |
| CD45 | Pan leukocyte marker | Mouse IgG1, clone X16/99 | Leica Biosystems, Buffalo Grove, Illinois, USA | 800 | 0·08 |
| CD68 | Macrophages/monocytes | Mouse IgG3, clone PG-M1 | Agilent (Dako), Santa Clara, California, USA | 500 | 0·08 |
| CD8 | Cytotoxic T lymphocytes | Mouse IgG1, C8/144B | Agilent (Dako), Santa Clara, California, USA | 500 | 0·31 |
| Chymase | Mast cells | Rabbit polyclonal | Atlas antibodies, Bromma, Sweden | 8000 | 0·04 |
| Collagen 1 | Extracellular collagen 1 | Rabbit polyclonal | Abcam, Cambridge, UK | 400 | 2·50 |
| Cytokeratin | Epithelium | Mouse IgG1, clone AE1/AE3 | Leica Biosystems, Buffalo Grove, Illinois, USA | 600 | 0·24 |
| Podoplanin | Lymphatic vessels | Mouse IgG1, clone D240 | Biocare Medical, Pacheco, California, USA | 50 | NA |
| ECP | Eosinophils | Mouse IgG1, clone EG2 | Diagnostics development, Upsalla, Sweden | 300 | 3·03 |
| glycophorin A | Erythrocytes | Rabbit monoclonal, clone EPR8199 | Abcam, Cambridge, UK | 100 | 1·99 |
| Ki-67 | Proliferation marker | Mouse IgG1, clone MIB-1 | Agilent (Dako), Santa Clara, California, USA | 100 | 0·46 |
| Langerin | Langerin+ dendritic cells | Mouse IgG2b, clone 12D6 | Leica Biosystems, Buffalo Grove, Illinois, USA | 100 | NA |
| MPO | Neutrophils | Rabbit polyclonal | Agilent (Dako), Santa Clara, California, USA | 20000 | 0·18 |
| Pro-MBP | Basophils | Mouse IgG1, clone J175-7D4 | Nordic Biosite, Täby, Sweden | 75 | 6·67 |
| Alpha-SMA | Smooth muscle | Mouse IgG2a, clone  1A4 | Agilent (Dako), Santa Clara, California, USA | 400 | 0·18 |
| Tryptase | Mast cells | Mouse IgG1, clone G3 | Nordic Biosite, Täby, SwedenMerck Millipore, Burlington, Massachusetts, USA | 12000 | 0·08 |
| Vimentin | Fibroblasts | Rabbit polyclonal | Abcam, Cambridge, UK | 200 | 5·00 |

CD, Cluster of differentiation; ECP, Eosinophil cationic protein; Ki-67, Marker of Proliferation; MOP, Myeloperoxidase; pro-MBP, pro-Major basic protein; SMA, smooth muscle actin; IgG, Immunoglobulin.

**Artificial Intelligence based spatial heterogeneity analysis of COVID-19 cases**

The spatial relationship between the multiple cell markers were analyzed by an artificial intelligence (AI)-based point pattern (i.e. x,y coordinate-based) approach by Stoltzfus et al.^3^ as well as a marker density-based heat map analysis..In order to generate proper x,y coordinates from densely clustered populations with fused cell marker objects the function Distance Transform Watershed (FIJI - ImageJ) was applied on each binary mask corresponding to the different dyes to separate touching cells. X-Y coordinates of cells were obtained with the function “Analyze Particles” (FIJI - ImageJ) and then used as input for the CytMAP Toolbox^3^. We used the function “Raster Scan Neighborhoods'' for neighborhood analysis, finding the local composition of cells within a circular area in the tissue. We choose a radius of radius 500px ≃ 150 µm, considering the average long distance among cytokine effects calculated *in silico* cell populations^4^. The function “Classify Neighborhoods into Regions'' was applied to define different tissue regions with similar immune cell composition. The classification was done using the artificial neural networks Self Organizing Map and the number of cell constellation classes was automatically defined as the minimum of the Davies-Bouldin function.

For the density-based approaches, the digitized whole section images were overlaid with a square raster of 1000 x 1000 pixel squares (typically around 700-1200 squares/section). From the SQL database the density of each marker was extracted from each tissue-containing square and the spatial correlations between different cell types was plotted as correlation matrix of different cell phenotypes using a custom MATLAB code exploiting the functions *corrcoef* and *clustergram.*

**In-Depth Profiling of Immune Cell and Structural Alterations Across DAD Patterns of COVID-19 cases**

A detailed spatial microenvironment analysis was applied to generate detailed insight into the immune cell signatures and structural alterations at distinct patterns of DAD. In brief, we carefully screened all standard hematoxylin-stained sections for regions of interest (ROI) with a homogeneous and uniform histopathological DAD pattern. In total, we identified 95 ROIs fulfilling these criteria. Next, high-resolution images from each region were classified into the patterns of DAD (acute DAD, Intermediate DAD, and advanced DAD). In parallel, the corresponding ROIs from the consecutive neighboring multiplex-stained sections were outlined and decoded for cell marker densities and cell composition analysis using the stored histomic raw database.

The generated DAD ROI data were subjected to basic statistics as well as multivariate analysis using a MATLAB-based principal component analysis (PCA) and K-means exploration to identify potential cluster formation in the PCA plot^4^. The markers having the most influence in the separation of DAD-associated clusters were identified by loading information.

**In Situ Hybridization of COVID-19 cases**

Visualization of SARS-CoV-2 and pan bacteria (16SrRNA) mRNA was performed through double *in situ* hybridization (ISH) using the RNAscope® Multiplex fluorescence kit V2 assay kit (#323100; Advanced Cell Diagnostics, Hayward, CA, USA) using the RNAscope® 2.5 HD Detection Reagents-RED (#322360, Advanced Cell Diagnostics) for chromogenic ISH (CISH), according to the manufacturer’s instructions.

Briefly, 4-µm lung tissue sections were baked at 60°C for 1h and then deparaffinized through a series of alcohol and xylene baths. Afterward, the slides were incubated with hydrogen peroxide for 10 min at RT and then with mRNA retrieval solution for 15 min at 99°C before dehydration with 99.5% EtOH for 3min at RT and then evaporation at 60°C for 5 min. After creating a hydrophobic border around each tissue specimen, they were incubated at 40°C for 30 min together with RNAscope Protease Plus and then at the same temperature for 2 h with the SARS-Cov-2 and 16SrRNA mRNA specific probes (#486731; Advanced Cell Diagnostics). Three controls were run in parallel: one negative control probe (DapB mRNA, #310043; Advanced Cell Diagnostics) and two positive control probes (PPIB, #313901; TPSAB1, #486081; Advanced Cell Diagnostics). Amplification and detection probes were then step-wise incubated with the samples, according to instructions. For chromogenic read-out, the red and chromogen included in the aforementioned RNAscope® 2.5 HD kit was used. The slides with chromogenic read-out were counterstained with Mayer’s hematoxylin (#01820; Histolab, Askim, Sweden) for 45 sec, before rinsing in dH_2_O and incubation in saturated LiCO_3_ for 5 sec, another rinsing step in dH_2_O and subsequent evaporation at 60°C for 20 min before mounting with pertex (#00840; Histolab) and No.1 coverslips (ECN 631-1574; VWR, Radnor, PA, USA).

**xMAP Luminex Cytokine Multiplex Assay of COVID-19 cases**

Samples of frozen lung tissue of one postmortem biopsy per case of the COVID-19 patients were prepared using Bio-plex Cell Lysis Kit (Bio-rad Laboratories, Hercules, CA, USA), following the manufacturer’s instructions. There was no availability of frozen tissue from control individuals. Cytokines and chemokines were assessed using the following kits: Bio-plex TGF-β, Bio-plex Human Chemokine (Bio-rad, Hercules, California) and Milliplex MAP Human Cytokine/Chemokine (Millipore Corp., Billerica, MA) according to the kit-specific protocols. Measurements were made using a Magpix analytical test instrument, which utilizes the xMAP technology in a multiple analytes profiling, and XPONENT 4.2 software and Milliplex Analyst 5.1 software (Millipore Corp., Billerica, MA, USA) was used for data analysis. To standardize the results, the total proteins of the lung samples were quantified with the TP2 kit (Roche diagnostics GmbH, Mannheim, Germany) using a COBAS C11 equipment (Roche Instrument Center, Rotkreuz, Switzerland). Data was expressed in pg/g and was correlated with clinical and pathological parameters.

**Nucleic Acids extraction of COVID-19 cases**

DNA and RNA were extracted from 80μm of FFPE lung samples. Briefly, the samples were incubated for 3h at 56ºC in 500μL of lysis buffer and 100 mg/mL of proteinase K (Qiagen, Hilden, Germany). Then, DNA and RNA were extracted using the Magna Pure Compact Nucleic Acid Isolation kit (Roche Diagnostics GmbH, Mannheim, Germany), according to the manufacturer’s instructions.

**Real-time reverse-transcription PCR of COVID-19 cases**

The viral load of SARS-CoV-2 was assessed by an in-house real-time PCR assay that amplifies part of the envelope protein gene, using conditions described previously in Corman et al, 2020. Positive and negative controls were included in all amplification reactions and the detection of the albumin gene was used as an internal control. The real-time PCR reactions were performed using the StepOne System equipment (Applied Biosystems, Foster City, CA, USA) following the program: 10 minutes at 95ºC, 45-50 cycles of 15 seconds at 94ºC and 60 seconds at 60ºC^5^. Primers and probes sequences are presented in Table S2.

**Table S2.** Primer and probe sequences used in the study

| **Target** | **Primer/Probe sequence** | |
| --- | --- | --- |
| Albumin | Forward | GTCAGCGTTCGTGTTTCCCA |
|  | Probe | VIC-CCCGCAACCCGCAACCCTTCATG- NFQ |
|  | Reverse | GGGACACAACACCGTAAAGC |
| Sars-Cov-2  Envelope gene | Forward | ACAGGTACGTTAATAGTTAATAGCGT |
|  | Probe | FAM-ACACTAGCCATCCTTACTGCGCTTCG-QSY |
|  | Reverse | ATATTGCAGCAGTACGCACACA |
| 5.8S rDNA-ITS  (outer primers) | Forward | TCCGTAGGTGAACCTGCGG |
|  | Reverse | TCCTCCGCTTATTGATATGC |
| 5.8S rDNA-ITS  (inner primers) | Forward | GCATCGATGAAGAACGCAGC |
|  | Reverse | TCCTCCGCTTATTGATATGC |
| 16S rDNA | Forward | TCCTACGGGAGGCAGCAGT |
|  | Reverse | GGACTACCAGGGTATCTAATCCTGTT |
| Glyceraldehyde 3-phosphate dehydrogenase (GAPDH) -outer primers | Forward | GACAACAGCCTCAAGATCATC |
|  | Reverse | GACGGCAGGTCAGGTCCACCA |
| Glyceraldehyde 3-phosphate dehydrogenase (GAPDH) -  inner primers | Forward | AATGCCTCCTGCACCACC |
|  | Reverse | ATGCCAGTGAGCTTCCCG |
| Peptidase inhibitor 3 (PI3) | Forward | CAGCTTCTTGATCGTGGTGG |
|  | Reverse | TGGACCTTTGACTGGCTCTT |
| Peptidoglycan recognition protein 1 (PGLYRP1) | Forward | CACATGAAGACACTGGGCTGGT |
|  | Reverse | CATGAAGCTGATGCCAATGGAC |
| Glycoprotein IX platelet (GP9) | Forward | CAGAGGAGAAGGCTGAGACC |
|  | Reverse | GGACTGAAGGCTGTTGTTGG |
| Immunoglobulin lambda variable 3-19 (IGLV3-19) | Forward | GGCCCCTGTACTTGTCATCT |
|  | Reverse | TAGTCAGCCTCATCTTCCGC |
| Collagen type III alpha 1 chain (COL3A1) | Forward | TGGATCAGGCCAGTGGAAAT |
|  | Reverse | AGTGTGTTTCGTGCAACCAT |
| Immunoglobulin heavy variable 1-58 (IGHV1-58) | Forward | TGCAAGGCTTCTGGATTCAC |
|  | Reverse | CATGTAGGCTGTGCTTGTGG |
| Ribosomal Protein S14 (RPS14) | Forward | GACTGGTGGGATGAAGGTAAAG |
|  | Reverse | TGATGTGTAGGGCGGTGATAC |

**Fungi and Bacteria detection in COVID-19 cases**

For the detection of pathogenic fungi, conventional nested PCR were performed in all samples with specific primers for the 28S to 18S interval regions of the rRNA gene, using conditions as described in Fujita et al.^6^. For the detection of several bacteria pathogenic to humans, conventional PCR was performed in all samples with specific primers for the 16S rDNA. The presence of amplifiable 16SrDNA was confirmed by PCR as described by Deutch et al.^7^. Primer sequences are presented in Table S2. All amplification reactions were performed using a Veriti 96 thermocycler (Applied Biosystems, Life Technologies Corporation, Carlsbad, CA, USA) and all PCR reagents were obtained from Invitrogen (Carlsbad, CA, USA). Positive, negative and endogenous controls were included in all assays.

For the endogenous control, the presence of amplifiable DNA was confirmed by nested PCR of a fragment of human glyceraldehyde-3-phosphate dehydrogenase (GADPH; GenBank: J04038.1), as described previously by Ercolani et al.^8^. For the fungal positive control, *H. capsulatum* ATCC A811 and B923, *C. neoformans* ATCC 24067, *P. brasiliensis* 18 and 339, *Aspergillus spp, Candida albicans* and *C. parapsilosis* samples were obtained from previous studies were amplified. For the bacteria's positive control, *Escherichia coli* (JM109) and *Pseudomonas aeruginosa* (ATCC 15442) samples obtained from previous studies were amplified. PCR products were examined for positivity and specificity via 1.5% agarose gel electrophoresis and then purified with PureLink Kit (QIAGEN, Hilden, Germany).

Samples of lung tissue that were positive for fungi and bacteria were submitted to sequencing analysis according to the Sanger method. Sequencing was performed using the kit ABI PRISM BigDye Terminator Cycle Sequencing Ready Reaction (Applied Biosystems, Life Technologies, Carlsbad, California, United States) according to the manufacturer’s instructions and the Genetic Analyzer ABI 3130 (Applied Biosystems, Life Technologies, Carlsbad, California, U.S). The sequences were analyzed using Sequencher 3.0 Sequencing Software (Ann Arbor, Michigan, United States) and compared with the genomic database GenBank (National Center for Biotechnology Information—NCBI- Bethesda, Maryland, U.S).

**Transcriptomics**

Twelve out of the 18 COVID-19 cases had adequate RNA quality of the frozen lung tissue to perform the transcriptomic analyses. Four control cases of individuals that died due to non-pulmonary causes and had frozen tissue of adequate quality were selected. Characteristics of the control individuals used for this transcriptomic analysis are presented in Table S3. The data presented in this publication have been deposited in NCBI's Gene Expression Omnibus and are accessible through GEO Series accession number GSE205099 (https://www.ncbi.nlm.nih.gov/geo/query/acc.cgi?acc=GSE205099).

**Table S3.** Demographic characteristics of control patients of the transcriptomic analyses.

| **Control Cases** | **Sex** | **Age (years)** | **BMI** | **Comorbidities** |
| --- | --- | --- | --- | --- |
| C1 | Female | 78 | 27 | Systemic arterial hypertension  Chronic renal disease  Cardiomyopathy |
| C2 | Male | 70 | 24·1 | Diabetes mellitus  Systemic arterial hypertension |
| C3 | Female | 77 | 21·1 | Cardiomyopathy  Smoker |
| C4 | Female | 46 | 23·1 | Systemic arterial hypertension  Adrenal Cancer |

RNA was extracted using RNeasy Mini Kit (Qiagen, Hilden, Germany) according to the manufacturer’s protocol followed by a DNAse treatment with Turbo DNAse Kit (Thermo Fisher Scientific, Waltham, MA, USA) at 37°C for 30 min. The concentration was determined using the Qubit® RNA HS Assay Kit (Thermo Fisher Scientific, Waltham, MA, USA) and the quality was analyzed using the Agilent 2200 TapeStation System with the RNA ScreenTape assay (Santa Clara, CA, USA). For RNA-sequencing (RNA-Seq), libraries were prepared using Illumina® RNA Prep with Enrichment kit, nt, IDT® for Illumina® DNA/RNA UD Indexes Set A, Tagmentation (Illumina, San Diego, CA, USA) and Illumina Exome Panel – Enrichment Oligos Only (Illumina, San Diego, CA, USA). Libraries were validated by quantification using Qubit dsDNA HS Assay kit (Invitrogen, Thermo Fisher Scientific, Waltham, MA, USA) and size measurement using Agilent 2200 TapeStation System with the High Sensitivity DNA ScreenTape assay. Sequencing was performed on the Illumina NovaSeq 6000 system to generate 150-bp paired-end reads.

All sequencing files were analyzed with FastQC (v0.11.9) [Babraham Bioinfomatics] to check the quality of the sequencing and look for adapters and other contamination in the sequences. After that, all reads were mapped to the human genome available in the ENSEMBL Database (GRCh38.p13 release 104)^9^ using STAR (v.2.7.6a)^10^ in two-pass mode using the following parameters: “--outFilterType BySJout --outFilterMultimapNmax 20 --alignSJoverhangMin 8 --alignSJDBoverhangMin 1 --outFilterMismatchNmax 999 --alignIntronMin 20 --alignIntronMax 1000000 --alignMatesGapMax 1000000 --outSAMstrandField intronMotif --outFilterIntronMotifs RemoveNoncanonicalUnannotated”.

After getting the coordinates of the read alignment in the human genome, transcript abundance was estimated using RSEM (v1.3.0)^11^ using default parameters. Transcript expression was then summarized to gene-level count using tximport (v.1.20.0)^12^ and then imported to DESeq2 (v.1.32.0)^13^ to perform differential expression analysis. Genes were classified as differentially expressed if |log fold change| > 0 and the false discovery rate was ≤ 0.05. Enrichment functional analysis [one based on the Gene Ontology (GO) and another based on KEGG pathways] of the differentially expressed genes (DEGs) was performed with clusterProfiler (v.4.0.0) ^14,15^ using genome annotations from Genome-wide annotation for Human (org.Hs.eg.db) R package (v3.13.0). GO terms and KEGG pathways were considered enriched if the adjusted p-value ^16^ was ≤ 0.05. Principal component analysis and volcano plots were generated using ggplot2 (v.3.3.3) and plotly (v.4.9.3) R packages. The heatmap plot was generated using gplots R package (v3.1.1).

We used real-time PCR to validate the differential expression of six candidate genes. These selected genes were chosen among the most differentially expressed genes within the enriched pathways relevant in the pathophysiology of COVID-19/DAD. Real-time PCR was conducted using 7500 Fast Real-Time PCR System (Applied Biosystems, Waltham, MA, USA) in the presence of SYBR-green SYBR Green PCR Master Mix (Applied Biosystems, Foster City, CA, USA).

Specific primers were designed using the program Primer3 version 4.1.0 to the following genes: PI3 (peptidase inhibitor 3), PGLYRP1 (peptidoglycan recognition protein 1), GP9 (glycoprotein IX platelet), PF4 (platelet factor 4), ADAM-12 (ADAM metallopeptidase domain 12), IGLV3-19 (immunoglobulin lambda variable 3-19), COL3A1 (collagen type III alpha 1 chain) and IGHV1-58 (immunoglobulin heavy variable 1-58). Primer sequences are described in Table S2.

Reverse-transcribed cDNA samples were performed using HighCapacity cDNA Reverse Transcription (Applied Biosystems, Foster City, CA, USA). The optimization of the real-time PCR reaction was performed according to the manufacturer's instructions but scaled down to 25 μl, followed by incubation at 95°C for 10 min, and 40 cycles of temperature varying from 95°C for 15 s to 60°C for 1 min. For all investigated transcripts three biological replicates were performed. The relative quantification of the transcripts was calculated after normalization against the levels of the reference gene RPS14 Ribosomal Protein S14 (RPS14).

**Table S4:** Individual values of the histological patterns and findings (%), cytokines assessed in lung tissue of COVID-19 patients (pg/g), viral load quantification (virus copies/100.000 cells), fraction of the firm tissue containing 16SrRNA mRNA, bacteria and opportunistic fungal infection identification.

**Table S5:** Correlations between tissue parameters with lung cytokines, chemokines and clinical data. Only significant associations are shown.

| **Tissue**  **Parameters** | **Correlations** |
| --- | --- |
| **Macrophages** | IL-1β (r=0·541, p=0·03), IL-10 (r=0·509, p=0·044), IFN-α2 (r=0·719, p=0·004), IFN-γ (r=0·576, p=0·019), MIP-3/CCL20 (r=0·524, p=0·037), TGF-β1 (r=0·597, p=0·015), TGF-β2 (r= 0·609, p=0·015) |
| **CD4+ T cells** | MIP-3/CCL20 (r=0·562, p=0·024), MDC/CCL2 (r=0·532, p=0·034), TARC/CCL17 (r=0·509, 0·044), IP-10 (r=0·609, p=0·012), MIG/CXCL9 (r=0·600, p=0·014) |
| **CD8+ T cells** | IL-1β (r=0·538, p=0·013), IL-4 (r=0·632, p=0·031), IL-6 (r=0·659, p=0·006), IL-8 (r=0·632, p=0·009), IL-10 (r=0·603, p=0·013), TNF-α (r=0·606, p=0·006), IFN-γ (r=0·738, p=0·001), MIP-3/CCL20 (r=0·691, p=0·003), TGF-β1 (r=0·671, p=0·004), TGF-β2 (r=0·559, p=0·024), TGF-β3 (r=0·541, p=0·03), MDC/CCL2 (r=0·656, p=0·001), TARC/CCL17 (r=0·635, p=0·008) |
| **Ki67+ cells** | IL-1β (r=0·556, p=0·025), IL-4 (r=0·503, p=0·047), IL-10 (r=0·524, =0·037), IFN-α2 (r=0·618, p=0·019), IFN-γ (r=0·05, p=0·049), TGF-β1 (r=0·703, p=0·002), TGF-β2 (r=0·618, p=0·011) |
| **CD31+ cells** | TGF-β1 (r=0·615, p=0·011) |
| **Langerin DC** | Time from symptom onset to death (r=0·596, p=0·009), hospitalization length (r=0·653, p=0·003), MV length (r=0·639, p=0·004), Intensive care unit stay (r=0·627, p=0·005), Exudative DAD (%) (r=-0·519, p=0·027), viral load (r=-0·579, p=0·012) |
| **Myeloid DC** | Time from symptom onset to death r=0·836, p=≤0·0001), hospitalization length (r=0·812, p=≤0·0001), MV length (r=0·812, p=≤0·0001), ICU stay (r=0·826, p=≤0·0001), exudative DAD (%) (r=-0·504, p=0·033), viral load (r=-0·707, p=0·001) |
| **Exudative DAD (%)** | MV length (r=-0·51, p=0·031) |
| **Viral load** | Time from symptom onset to death (r=-0·727, p=0·001), period of hospitalization (r=-0·768, p=≤0·0001), MV length (r=-0·703, p=0·001), ICU stay (r=-0·789, p=≤0·0001), Exudative DAD (%) (r=0·609, p=0·007) |

IL-1β, Interleukin-1 beta ; IL-6, Interleukin-6; IL-8, Interleukin-8; IL-10, Interleukin-10; TNF-α, Tumour necrosis factor alpha; IFN-α2, Interferon alpha-2; IFN-γ, Interferon gamma; MIP-3/CCL20, Human CCL20/MIP-3 alpha Protein; TGF-β1,  Transforming growth factor beta 1; TGF-β2,  Transforming growth factor beta 2; TGF-β3, Transforming growth factor beta 3; MDC/CCL2, Macrophage-derived chemokine 2; TARC/CCL17, Thymus- and activation-regulated chemokine ligand 17; IP-10, Interferon gamma-induced protein 10; MIG/CXCL9, Monokine induced by gamma interferon chemokine ligand 9; DAD, Diffuse alveolar damage; DC, Dendritic cells; MV, Mechanical ventilation; ICU, Intensive Care Unit

**Table S6:** Total of reads obtained from RNA-Seq and the number of aligned reads to the human genome in each sample.

**Table S7:** List of DEGs obtained from differential expression analysis between exudative DAD and control, intermediate/advanced DAD and control, and the shared DEGs between both comparisons.

**Table S8:** DEGs expression scaled to Z-Score in each sample.

Note: The order of the rows in this table is the same order of the rows in the heatmap (Supplementary figure S8), thus, the top genes in the heatmap are also the top genes in this table.

**Table S9:** Enriched GO terms and KEGG pathways in the DEGs that are exclusive to the exudative DAD and control samples comparison. BP = biological process, MF = Molecular Function, CC = Cellular Component.

**Table S10:** Enriched GO terms and KEGG pathways in the DEGs that are exclusive to the intermediate/advanced DAD and control samples comparison. BP = biological process, MF = Molecular Function, CC = Cellular Component.

**Table S11:** Enriched GO terms and KEGG pathways in the DEGs that are shared between the exudative and intermediate/advanced DAD in comparison to control samples. BP = biological process, MF = Molecular Function, CC = Cellular Component.

**Supplementary Figures**

**
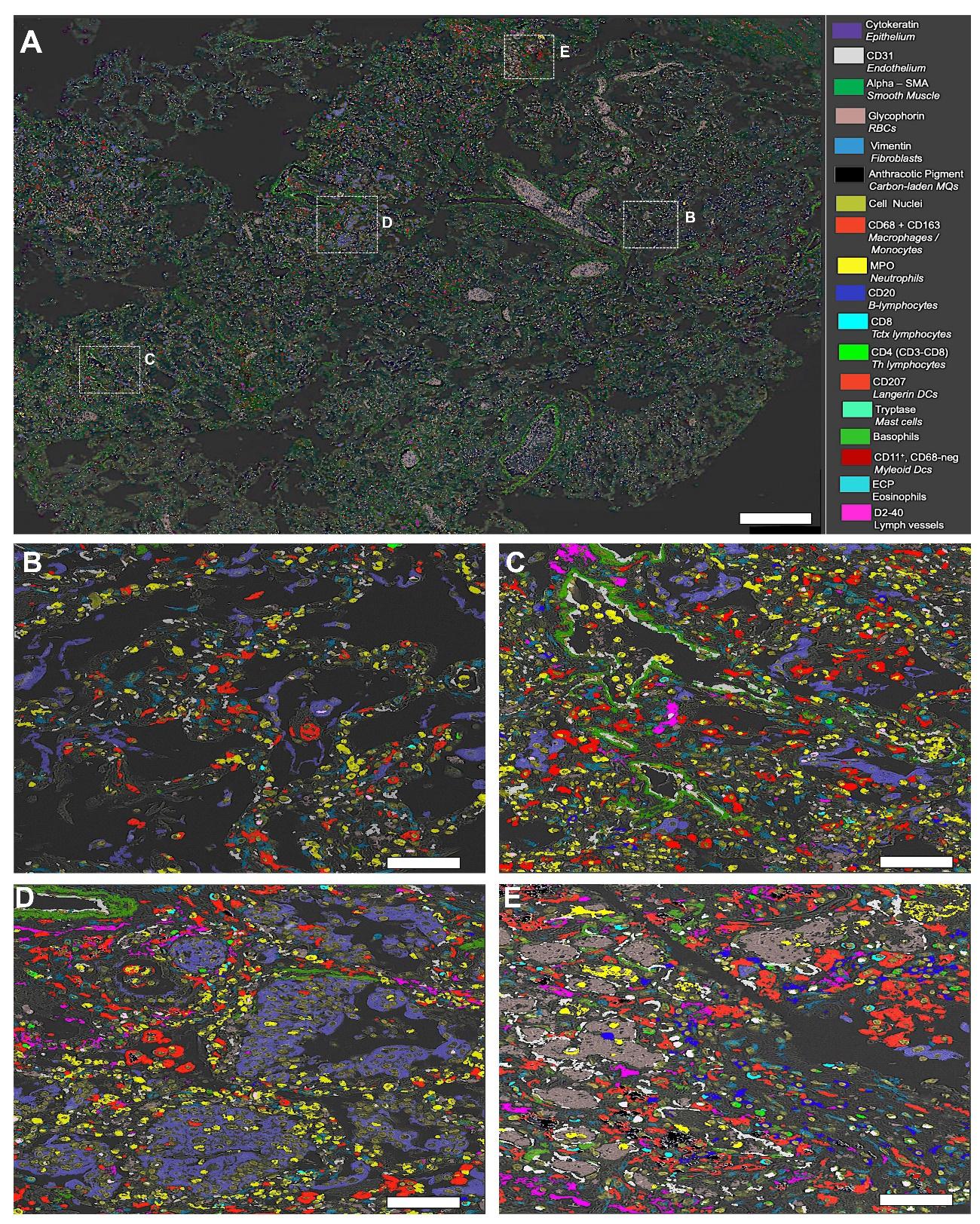
**

**Figure S1. Compartmentalized and patchy immunopathological patterns in COVID-19 affected lungs:** **Decoding of the spatial heterogeneity of structural and immunological cells by multiplex IHC.**

(A) Low power overview. (B) Zoomed-in region from A with exudative DAD and where the immune cell picture is dominated by macrophages and neutrophils. (C) Intermediate DAD. (D) Intermediate DAD with hyperplastic epithelium (blue) and, (E) advanced DAD. Note the emergence of lymph vessels and lymphocytes as DAD progresses into an advanced pattern. Scale bars: A = 0·3 mm; B and E = 50 µm; C and D = 60 µm.

**
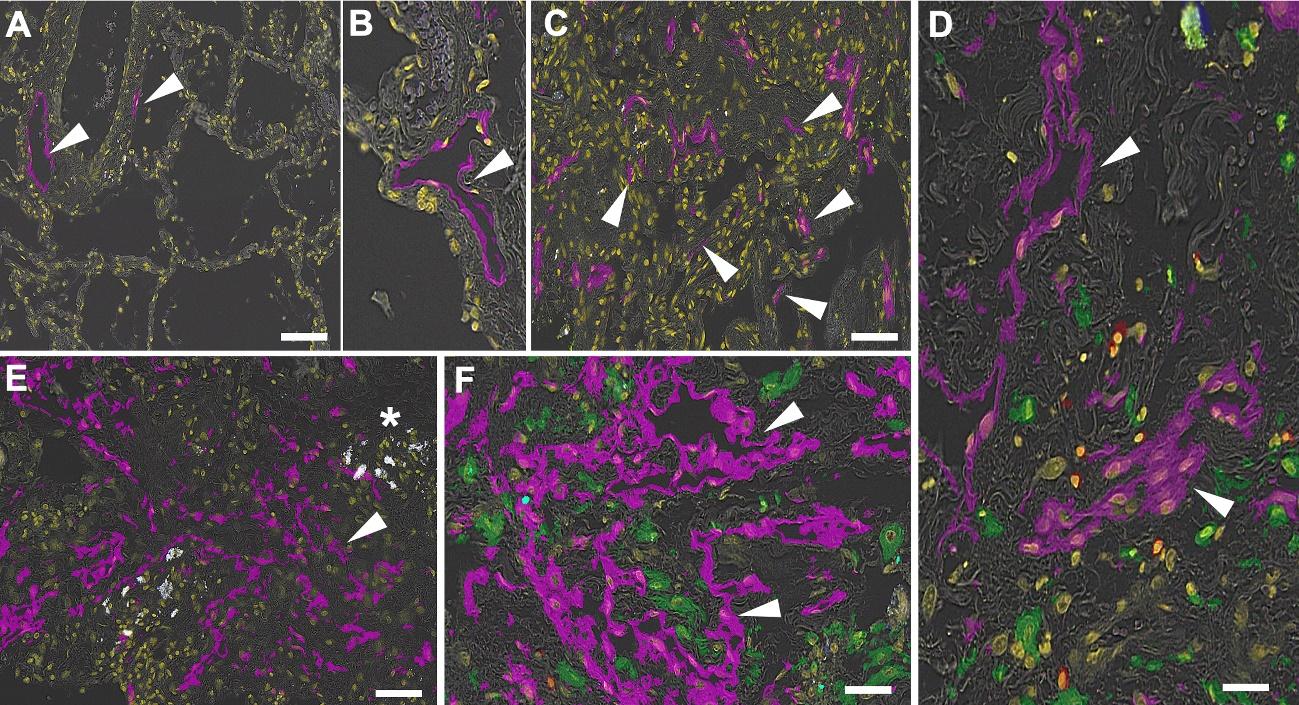
**

**Figure S2**. **Patchy expansion of lymphatic vessels in distal COVID-19 lungs.**

(A-B) Immunohistochemical identification of lymph vessels (D2-40-positivity) in a control lung. Emergence of new and smaller lymph vessels/lymph endothelial cells in COVID -19 lungs. (C) Area with abundant solitary D2_40-positive cells. (D-F) illustrate the more irregular shape of the distal lung lymphatics observed in COVID-19. Green cells in D and F are macrophages. White arrows exemplify the different morphological types of lymph vessels. Asterisk in E denotes macrophage with anthracotic pigment. Scale bars: A and E 100 µm; C = 80 µm; B and F = 50µm; D = 35µm.


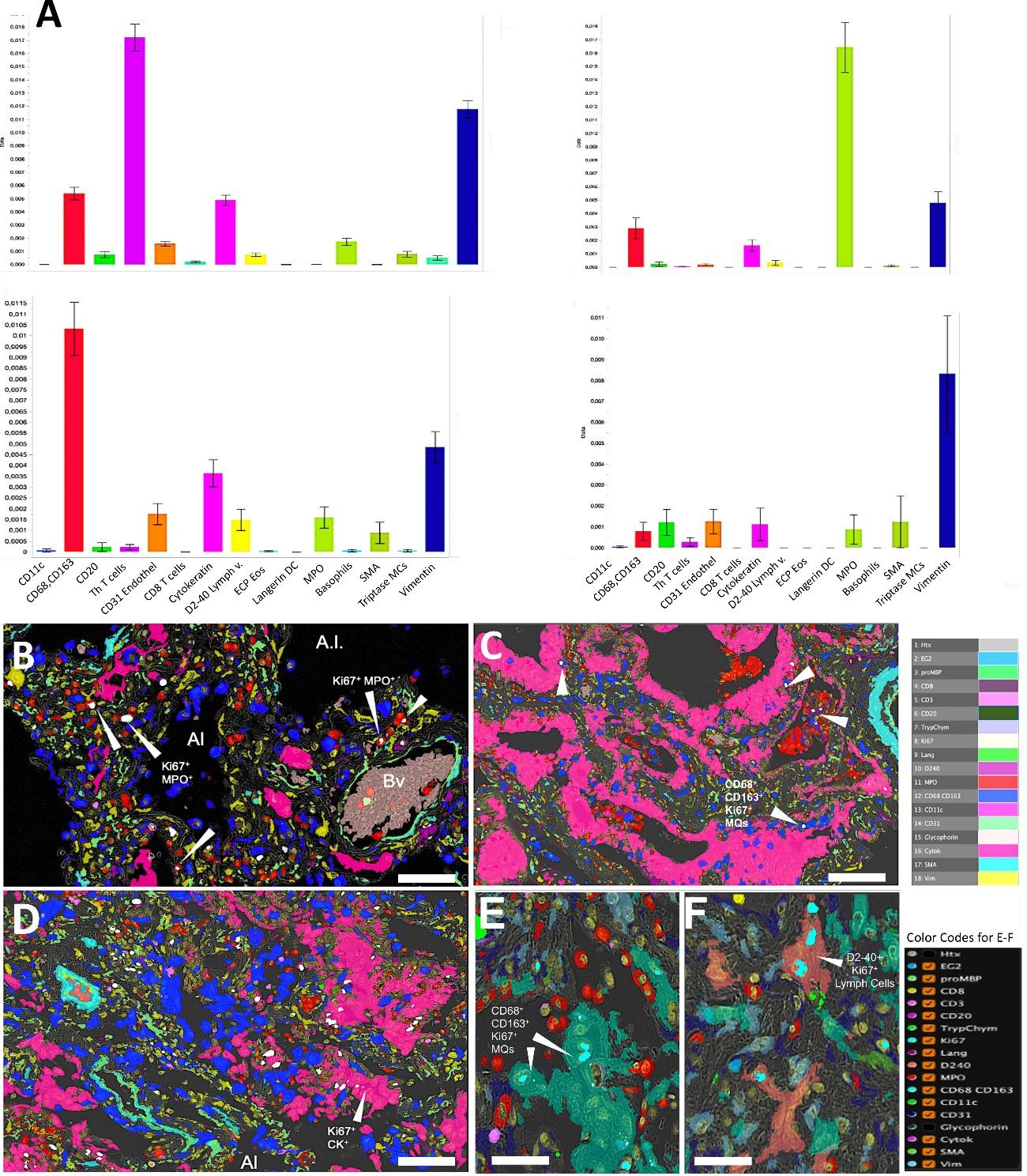


**Figure S3.** **Multifaceted and heterogeneous cell proliferation in COVID-19.**

(A) Example of relative cell expression of the proliferation marker Ki67 in four different subjects. Note the difference in both Ki-67 expression and the type of main Ki67-positive cell types. (B-F) Pseudo color-coded micrographs exemplifying Ki-67+ MPO-positive cells (red in B), Ki67-positive (white) CD68, CD163 macrophages (blue in C), proliferating hyperplastic epithelium (pink in D). Arrowheads in E and F show Ki-67 (turquoise) macrophages and lymphatic vessels, respectively. Al = alveolar lumen. Scale bars: B and D = 70 µm; C = 90 µm; E and F = 25 µm.

**
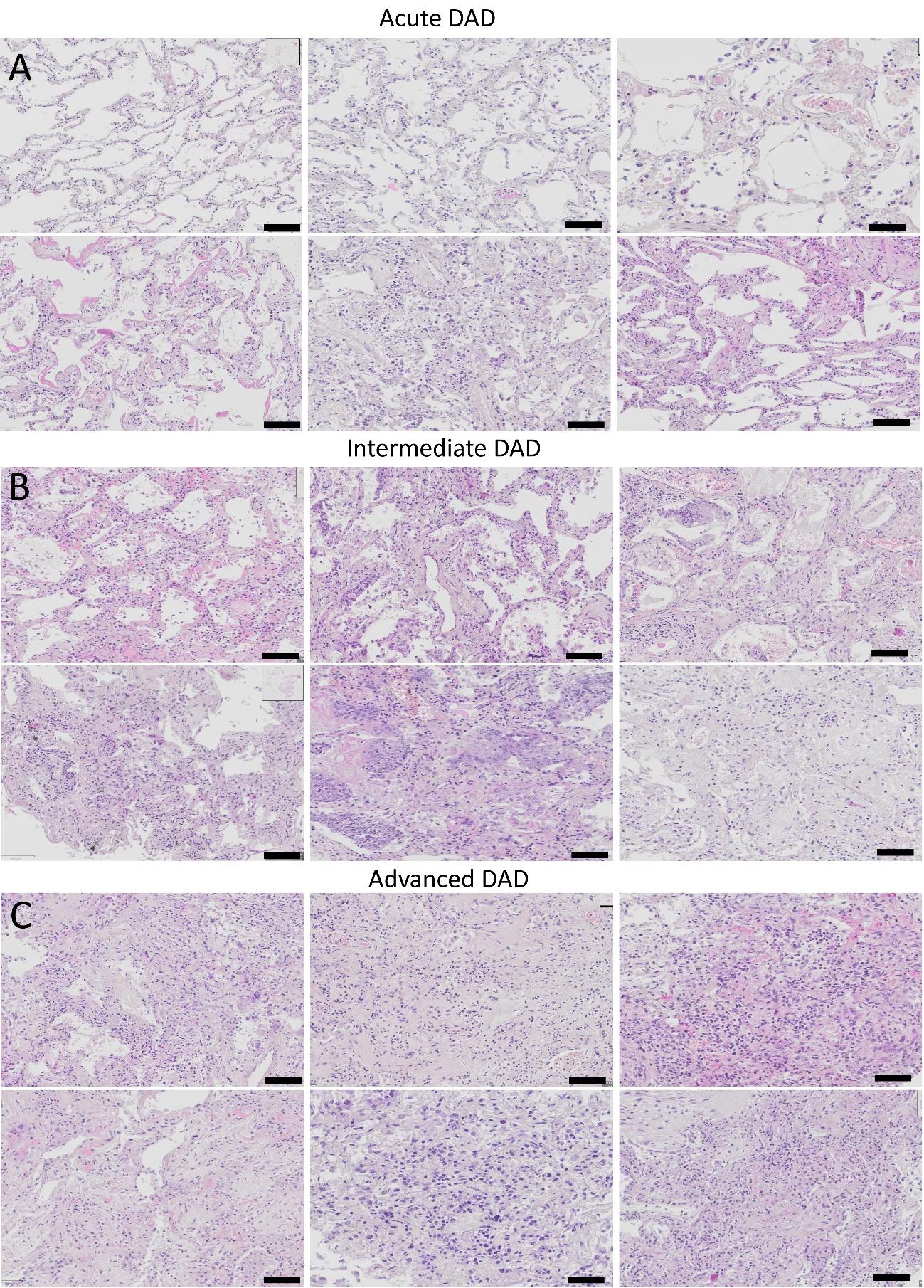
**

**Figure S4. Micrographs from hematoxylin-eosin-stained sections** **illustrate the basic histopathological features of acute, intermediate, and advanced DAD categories.** The individual panels are from distinct tissue regions of interest (ROIs) corresponding to exudative (A), intermediate (B), and advanced diffuse alveolar damage (DAD). Scale bars: All = 150 µm; except upper right in A, center up and down in B, and upper right, and center down in C where scale bars represent 100 µm.


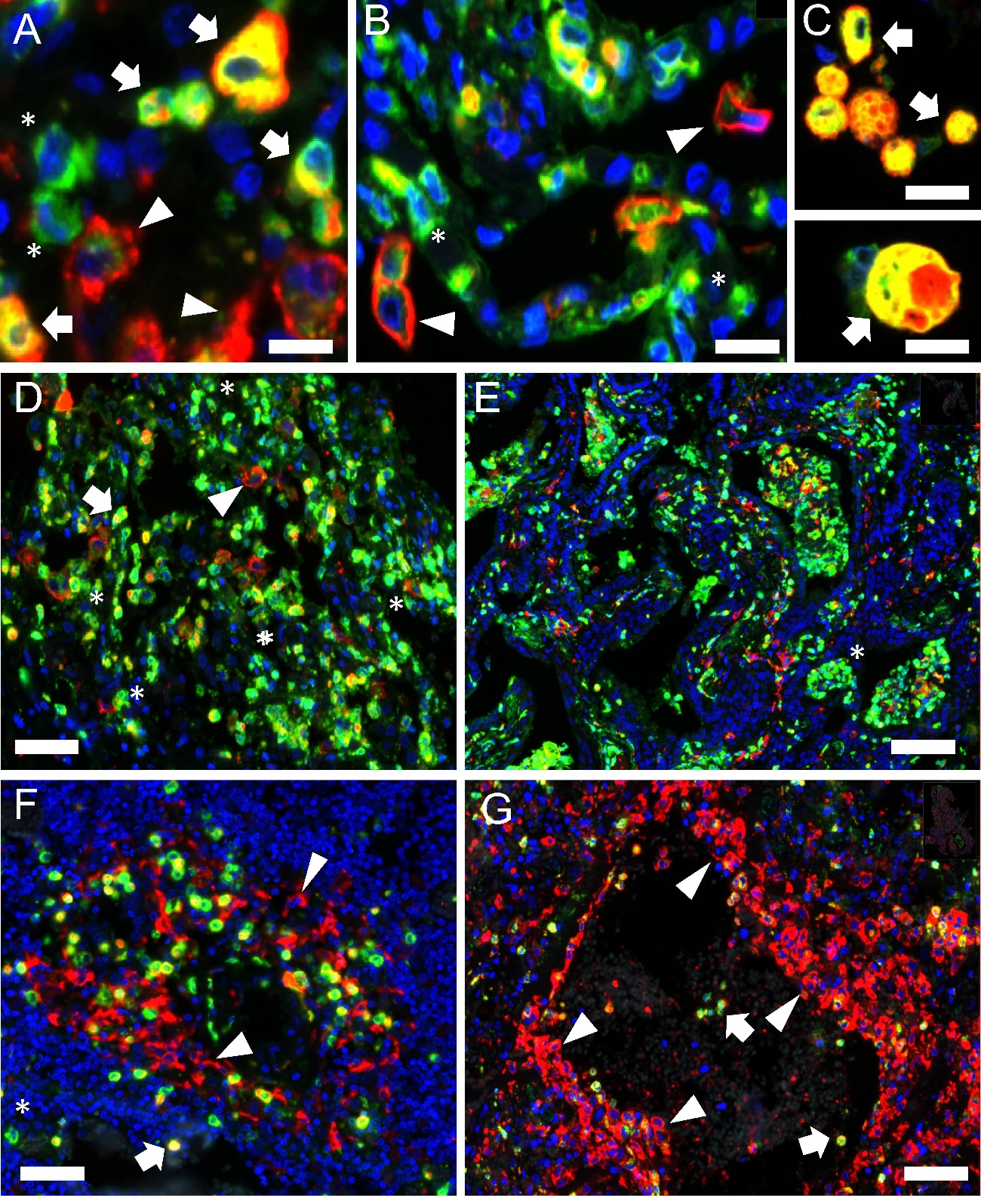


**Figure S5: Complex patterns of lysozyme-positive and CD206-positive macrophages in COVID-19 lungs samples.** (A-C) Immunofluorescence double-staining exemplifying the occurrence of single lysosome-positive cells (asterisks), single CD206-positive cells (arrowheads), and lysozyme+CD206 double-positive cells (arrows). Lysozyme immunoreactivity is green, whereas red denotes CD206 staining. Nuclei are stained blue by DAPI. (D) One of the rare tissue microenvironments in the present study material with high viral presence displaying a foremost lysozyme-positive monocyte/macrophage phenotype. (E) Area with intermediate DAD and high content of lysozyme-skewed luminal macrophages. (F) Region associated with lymphoid tissue and differentiated populations with lysozyme-skewed cells amidst larger irregularly shaped CD206-skewed macrophages. (G) Dense macrophage sheets where the macrophage phenotype is CD206-skewed. Scale bars: A= 20 µm; B= 30 µm; C = 35 µm (upper) 20 µm (lower); D, E, and G = 120 µm; F = 100 µm.

**
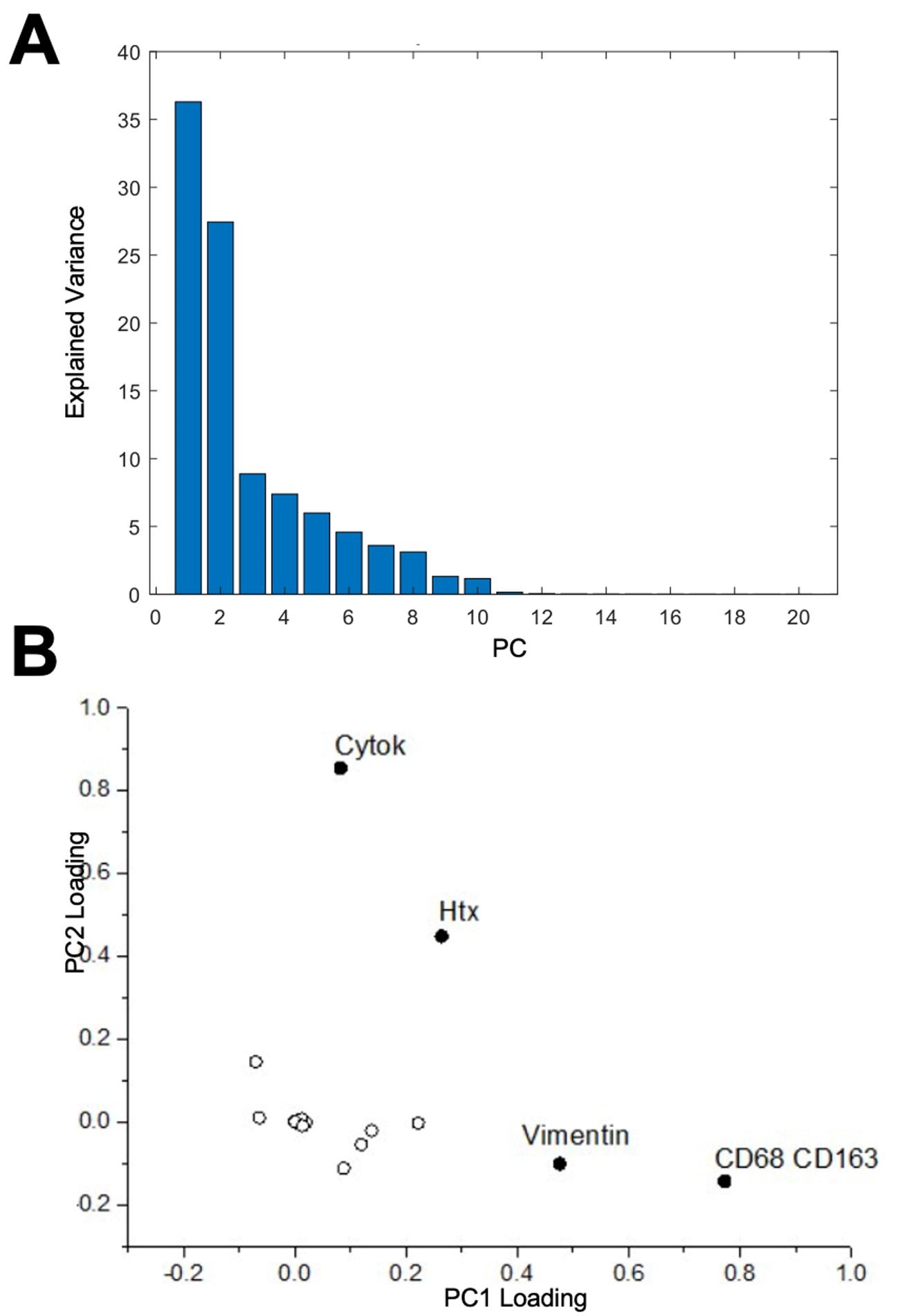
**

**Figure S6.** **Background data to support the analysis of DAD ROIs by principal component analysis**. (A) Bar plot showing the percentage of explained variances versus principal components (PCs). (B) Loading plot for the principal components PC1 and PC2 in Fig 4F revealing cytokeratin and overall cell density (Htx) as the most correlated with PC2, whereas CD68 and vimentin are strongly correlated with PC1.

**
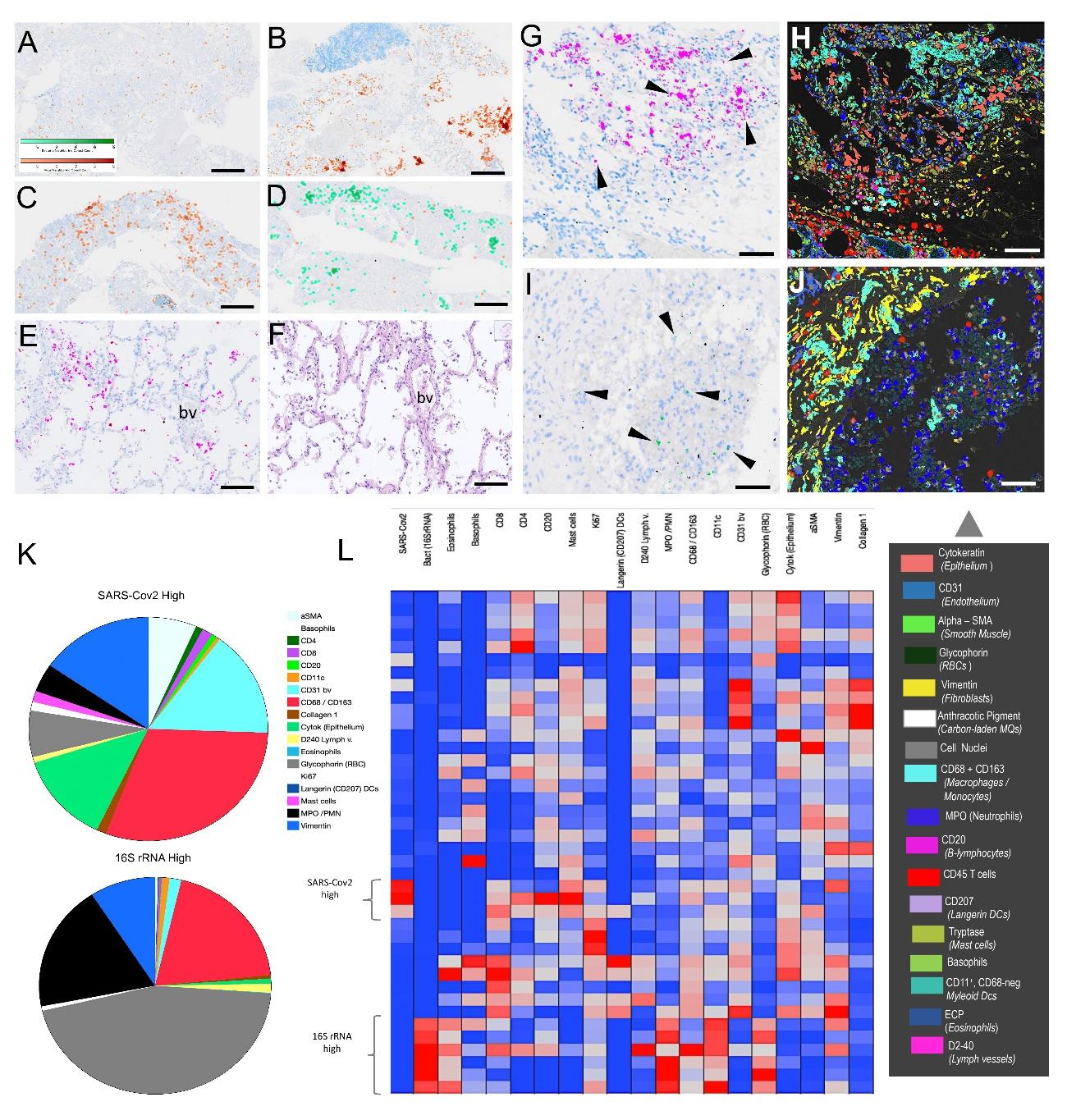
**

**Figure S7.** **Leukocyte and structural cell composition in the few observed microenvironments with significant SARS-CoV-2 or bacterial presence.**

(A-D) Low power overview images from double ISH-stained slides with artificially enlarged ISH dots representing SARS-CoV2 mRNA (orange-brown) and bacterial S16rRNA (green). (E-F) Zoomed in exudative DAD area with high SARS-CoV-2 (pink pseudocolored ISH dots) and corresponding area in H&E. (G-H). Tissue area with SARS-CoV-2 (arrowheads, left) and corresponding multiplex IHC image (right). (I-J) Area with bacterial S16rRNA (pseudocolored green ISH dots, left) with corresponding multiplex IHC image (right, color codes below). (K-L). Cell plot with relative abundance (high=red, low=blue) of multiplex IHC markers. Each row corresponds to one tissue region of interest (ROI). Pie charts of marker density proportions from the SARS-CoV-2 and 16SrRNA high ROIs (left). Bv = blood vessel. Scale bars: A-D = 0.5 mm; E and F = 100 µm; G and H = 60 µm; J and I = 40 µm.

**
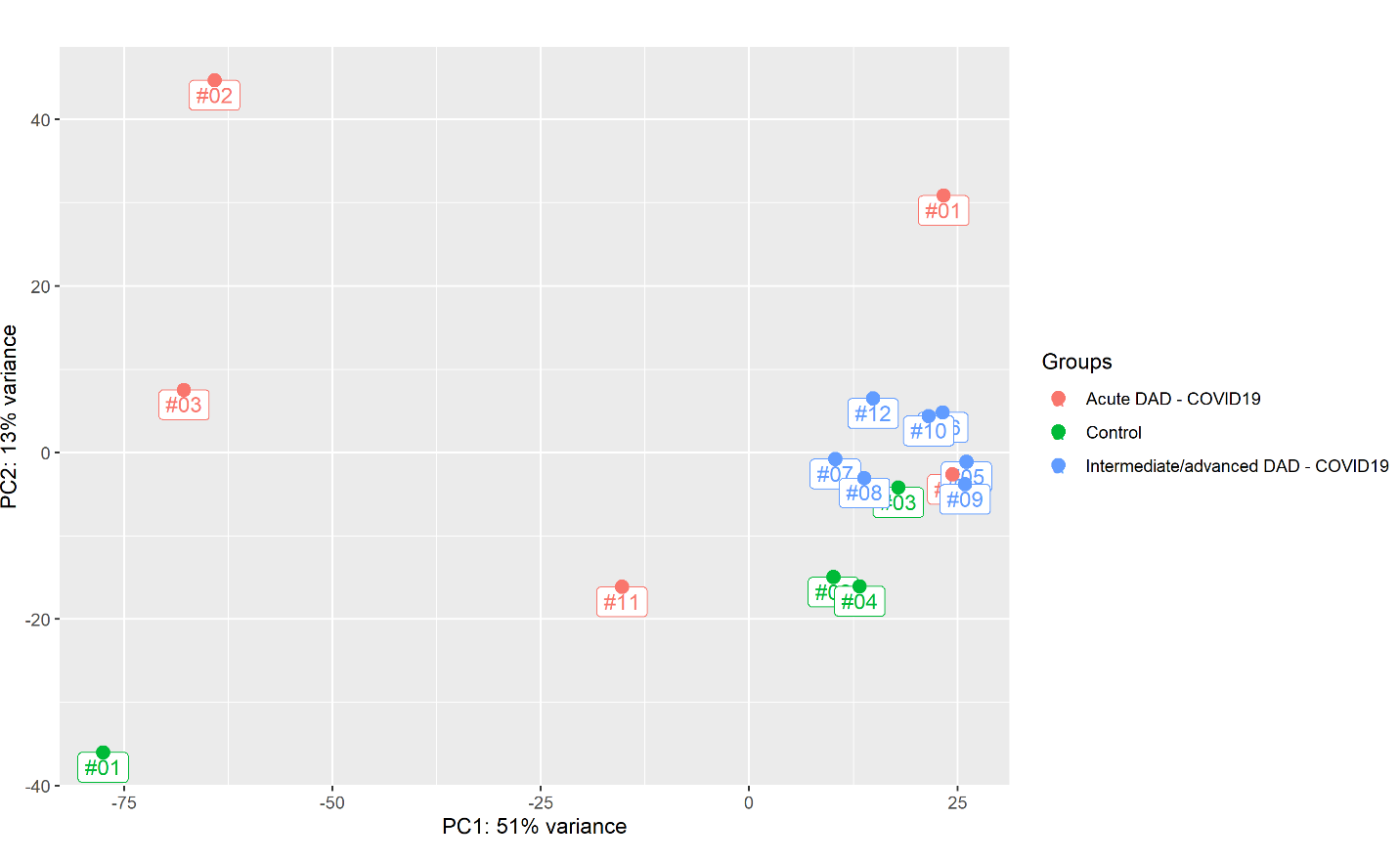
**

**Figure S8.** **PCA plot of the RNA-Seq samples.**

**
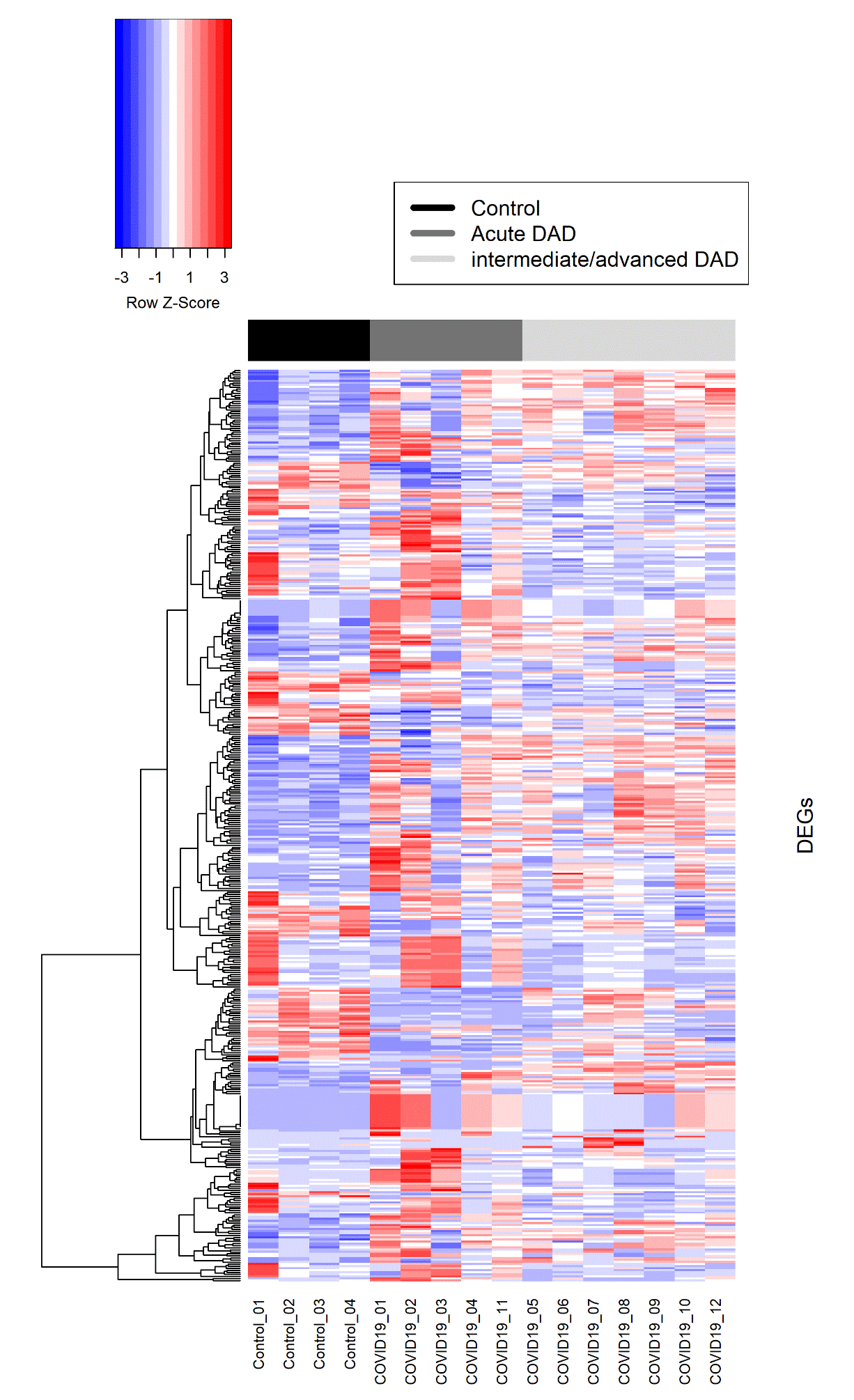
**

**Figure S9.** **Heatmap of the DEG profiles in each sample.**

**
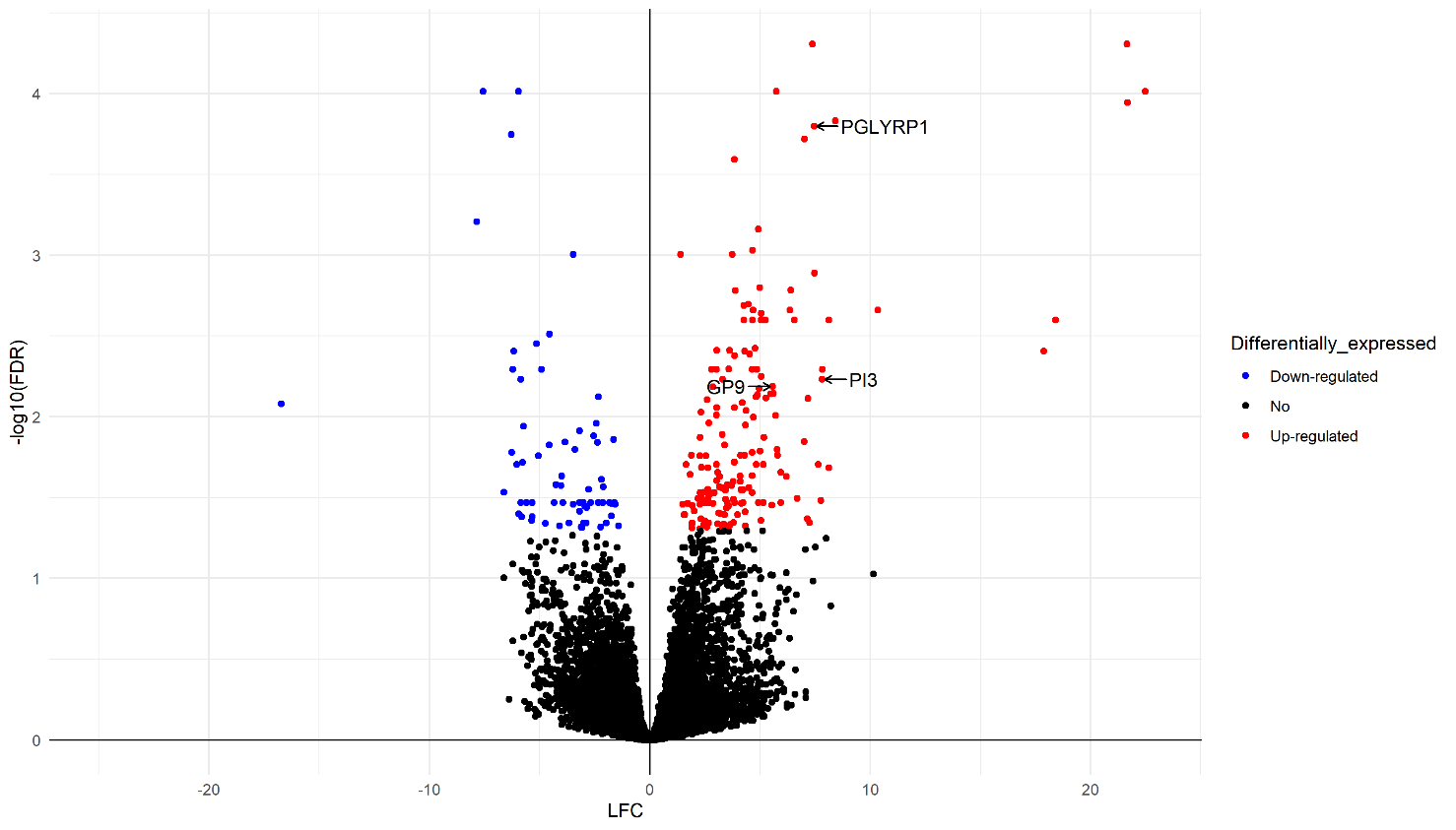
**

**Figure S10.** **Volcano plot of the results of differential expression analysis between exudative DAD and controls.** The highlighted genes were selected for validation by real-time PCR.

**
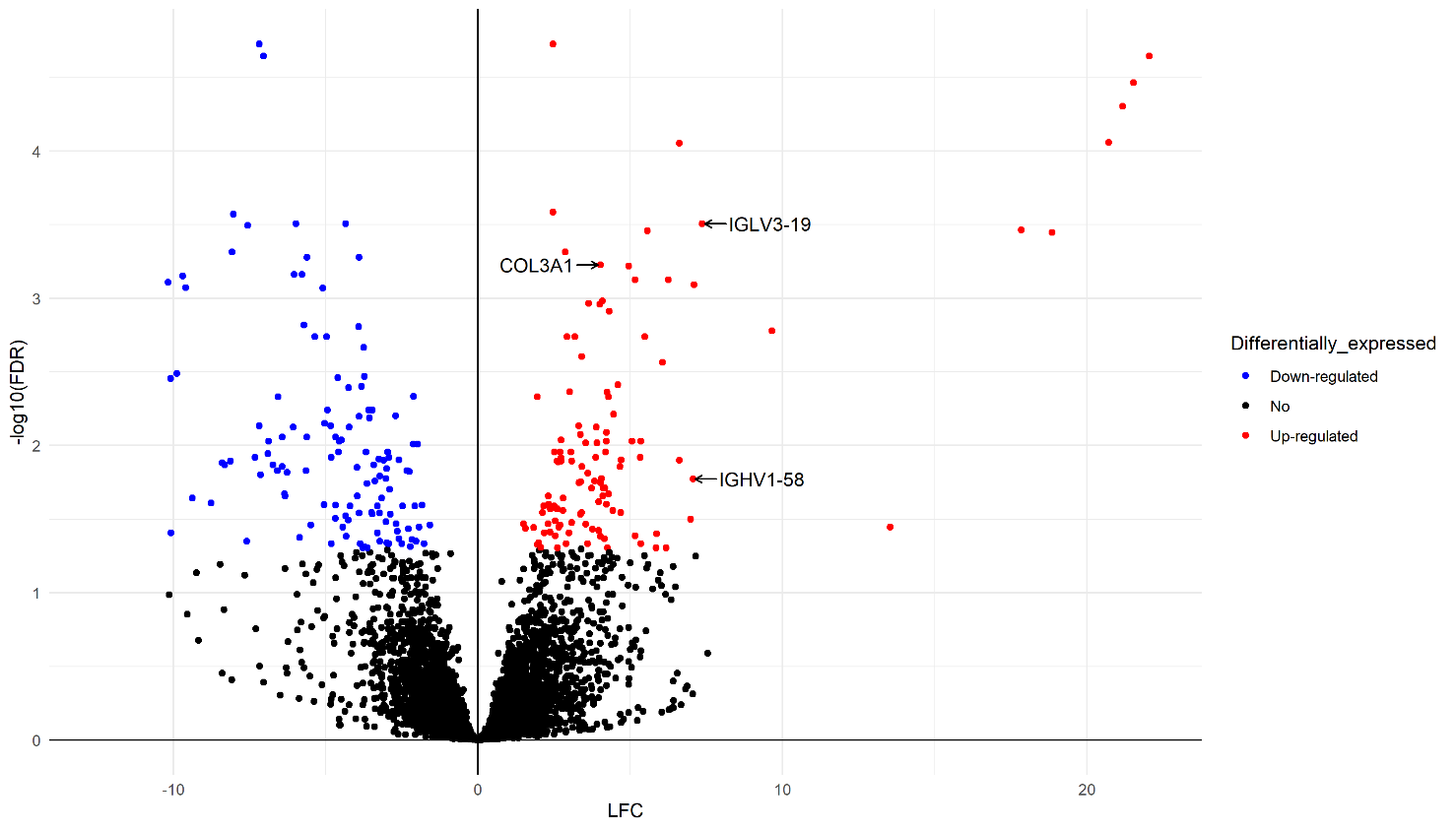
**

**Figure S11.** **Volcano plot of the results of differential expression analysis between intermediate/advanced DAD and controls.** The highlighted genes were selected for validation by real-time PCR.

**References**

1 Duarte-Neto AN, Monteiro RA de A, Johnsson J, *et al.* Ultrasound-guided minimally invasive autopsy as a tool for rapid post-mortem diagnosis in the 2018 Sao Paulo yellow fever epidemic: Correlation with conventional autopsy. *PLOS Neglected Tropical Diseases* 2019; **13**: e0007625.

2 Duarte‐Neto AN, Monteiro RAA, Silva LFF, *et al.* Pulmonary and systemic involvement in COVID‐19 patients assessed with ultrasound‐guided minimally invasive autopsy. *Histopathology* 2020; **77**: 186–97.

3 Stoltzfus CR, Filipek J, Gern BH, *et al.* CytoMAP: A Spatial Analysis Toolbox Reveals Features of Myeloid Cell Organization in Lymphoid Tissues. *Cell Reports* 2020; **31**: 107523.

4 Thurley K, Gerecht D, Friedmann E, Höfer T. Three-Dimensional Gradients of Cytokine Signaling between T Cells. *PLoS computational biology* 2015; **11**: e1004206.

5 Corman VM, Landt O, Kaiser M, *et al.* Detection of 2019 novel coronavirus (2019-nCoV) by real-time RT-PCR. *Eurosurveillance* 2020; **25**. DOI:10.2807/1560-7917.ES.2020.25.3.2000045.

6 Fujita S-I, Senda Y, Nakaguchi S, Hashimoto T. Multiplex PCR Using Internal Transcribed Spacer 1 and 2 Regions for Rapid Detection and Identification of Yeast Strains. *Journal of Clinical Microbiology* 2001; **39**: 3617–22.

7 Deutch S, Dahlberg D, Hedegaard J, Schmidt MB, Møller JK, Ostergaard L. DIAGNOSIS OF VENTRICULAR DRAINAGE-RELATED BACTERIAL MENINGITIS BY BROAD-RANGE REAL-TIME POLYMERASE CHAIN REACTION. *Neurosurgery* 2007; **61**: 306–12.

8 Ercolani L, Florence B, Denaro M, Alexander M. Isolation and complete sequence of a functional human glyceraldehyde-3-phosphate dehydrogenase gene. *The Journal of biological chemistry* 1988; **263**: 15335–41.

9 Howe KL, Achuthan P, Allen J, *et al.* Ensembl 2021. *Nucleic Acids Research* 2021; **49**: D884–91.

10 Dobin A, Davis CA, Schlesinger F, *et al.* STAR: ultrafast universal RNA-seq aligner. *Bioinformatics* 2013; **29**: 15–21.

11 Li B, Dewey CN. RSEM: accurate transcript quantification from RNA-Seq data with or without a reference genome. *BMC Bioinformatics* 2011; **12**: 323.

12 Soneson C, Love MI, Robinson MD. Differential analyses for RNA-seq: transcript-level estimates improve gene-level inferences. *F1000Research* 2016; **4**: 1521.

13 Love MI, Huber W, Anders S. Moderated estimation of fold change and dispersion for RNA-seq data with DESeq2. *Genome Biology* 2014; **15**: 550.

14 Wu T, Hu E, Xu S, *et al.* clusterProfiler 4.0: A universal enrichment tool for interpreting omics data. *The Innovation* 2021; **2**: 100141.

15 Yu G, Wang L-G, Han Y, He Q-Y. clusterProfiler: an R Package for Comparing Biological Themes Among Gene Clusters. *OMICS: A Journal of Integrative Biology* 2012; **16**: 284–7.

16 Benjamini Y, Hochberg Y. Controlling the False Discovery Rate: A Practical and Powerful Approach to Multiple Testing. *Journal of the Royal Statistical Society: Series B (Methodological)* 1995; **57**: 289–300.
